# Auto‐inducible expression of chimeric antigen receptor T cells using the NR4A1 promoter

**DOI:** 10.1111/imcb.70095

**Published:** 2026-03-08

**Authors:** Samuel WJ Smith‐Bell, Joshua C Halpin, Phillip K Darcy, Thiloma Liyanage, Lachlan J Dobson, Alexander D McLellan

**Affiliations:** ^1^ School of Medical Sciences, Faculty of Medicine and Health University of Sydney Camperdown NSW Australia; ^2^ Department of Microbiology and Immunology University of Otago Dunedin Otago New Zealand; ^3^ Children's Cancer Research Unit Children's Hospital at Westmead Sydney NSW Australia; ^4^ Cancer Immunology Program Peter MacCallum Cancer Centre Melbourne VIC Australia; ^5^ Sir Peter MacCallum Department of Oncology The University of Melbourne Parkville VIC Australia; ^6^ Department of Histology Faculty of Medicine, Universitas Indonesia Jakarta Indonesia

**Keywords:** CAR T cells, inducible promoters, PD1, T cell exhaustion

## Abstract

Chimeric antigen receptor (CAR) T cell therapies have shown remarkable efficacy in hematological malignancies, yet translation to solid tumors has been hindered by immunosuppressive tumor microenvironments, reduced T cell persistence and on‐target/off‐tumor toxicities. Constitutive CAR expression, typically driven by strong promoters such as EF1α, promotes tonic signaling, receptor clustering and antigen‐independent activation, contributing to T cell exhaustion and adverse events. Inducible promoter systems have been proposed to improve control over CAR expression. NR4A1, a transcription factor (TF) activated during early T cell receptor (TCR) signaling, governs pathways central to T cell activation and dysfunction, making its promoter an attractive candidate for conditional CAR regulation. We compared constitutive (EF1α), synthetic inducible (6NFAT‐NFκB and 2NFAT‐2NurRE) and NR4A1 promoters to drive expression of a second‐generation FRP5‐CAR. NR4A1‐driven CARs demonstrated low basal expression that was rapidly induced upon antigen encounter, reaching levels equivalent to EF1α‐driven CARs while showing minimal antigen‐independent signaling. Functionally, NR4A1‐driven CARs mediated potent tumor lysis, preserved a less exhausted (PD‐1^low^ and TIM‐3^low^) and more memory‐like phenotype (CD62L^high^ and CD45RA^high^), and sustained robust antitumor responses in vitro and in vivo. These findings establish the NR4A1 promoter as a native, activation‐inducible system to fine‐tune CAR expression, while maintaining therapeutic efficacy comparable to constitutively expressed CAR T cells. This strategy provides a promising framework for advancing CAR T cell therapies against solid tumors.

## Graphical abstract

The NR4A1 promoter is a compact auto‐inducible promoter to drive CAR expression in T cells. Compared with EF1α, or existing synthetic antigen‐inducible promoters, NR4A1 is more compact and ensures that resting CAR T cells express reduced baseline CAR. NR4A1 allows rapid induction upon antigen encounter with reduced T‐cell exhaustion.

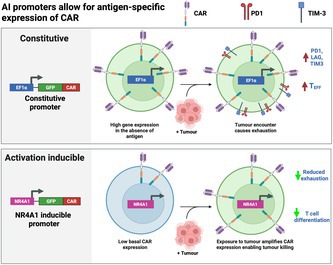

## INTRODUCTION

The engineering of patient lymphocytes to express tumor‐specific chimeric antigen receptors (CARs) has seen great efficacy in the treatment of B‐cell lymphomas and leukemias, such as relapsed or refractory acute B‐cell leukemia (ALL), diffuse large cell lymphoma (DLBCL), and multiple myeloma (MM).^1^, ^2^, ^3^ However, this success has had limited translation in targeting solid tumors. The application of CAR T cells in the treatment of solid tumors has been met with unique challenges, namely the presence of immunosuppressive tumor microenvironments (TME), which have been observed to reduce the persistence of CAR T cells as well as off‐tumor antigen recognition.^4^ The “on‐target off‐tumor” phenomenon has been attributed to the expression of high‐density, high‐affinity CARs on the T cell surfaces, triggering inappropriate immune activation resulting in adverse reactions, including cytokine release syndrome (CRS) and neurotoxicity.^5^, ^6^, ^7^, ^8^

The constitutive expression of CARs is at odds with the comparatively tighter control and regulation of TCRs naturally expressed by T cells; this discrepancy may offer an explanation for the lack of CAR persistence and increased exhaustive phenotypes observed in clinical trials.^9^, ^10^ Recently, the use of conditionally activated promoters has seen increased interest; among these promoters, “activation inducible” (AI) promoters that respond to the stimulation of T cells have been of particular interest in the field of CAR T cells for the controlled expression of stimulatory molecules, such as cytokines.^11^, ^12^, ^13^ These promoters are often composed of transcription factor binding sites, most commonly including NFAT, NFκB and AP1 binding sites spliced to minimal downstream promoter elements.^14^, ^15^, ^16^ While the use of these promoters has facilitated the fine‐tuned expression of accessory genes, there has been limited application of auto‐inducible promoters in refining the expression of the CAR itself.

Prior modifications to CAR expression cassettes to address the issues of persistence and safety have included alterations to the linker length, affinity tuning mutations of complementarity determining regions (CDRs) and the choice of promoters used for cassette expression.^17^, ^18^, ^19^, ^20^, ^21^ While effective linker or CDR residue modification is often specific to the CAR design, promoter choice can be more ubiquitously applied to various CAR designs.^22^, ^23^, ^24^ Our previous lab findings observed that CARs driven by EF1a increased CAR expression and increased target cell lysis; recent investigations have shown that reduced surface expression of CAR molecules increases the persistence of CAR T cells and promotes a less exhaustive (PD‐1^low^, TIM3^low^, LAG3^low^) and increased memory‐like (CD62L^high^ and CD45RA^high^) phenotypes which results in a more robust clinical response.^20^, ^25^, ^26^, ^27^, ^28^ This early exhaustion has been linked to antigen‐independent activation/stimulation, which occurs through receptor clustering on the cell surface, often due to over‐expressed transcript from constitutive expression.^23^

Here, we detail the use of the minimal NR4A1 promoter for the expression of a second‐generation CAR. In contrast to the synthetic promoters composed of TF DNA binding elements, the minimal Nr4a1 promoter is a native T cell promoter that controls the expression of early activation genes of the Nr4a family.^29^ The Nr4a1 family of genes has been labeled a key regulator of pathways intrinsic to T cell dysfunction. The family is composed of three proteins, NR4A1 (Nur77), NR4A2 (Nurr1) and NR4A3 (Nor‐1), respectively, which contain zinc‐finger DNA binding domains that bind to genes involved in T cell survival, proliferation, angiogenesis, inflammation and DNA repair.^29^ The NR4A1 promoter plays a critical role in the thymic selection of T cells via triggering NR4A1 expression via Ca^2+^ flux‐mediated TCR engagement.^30^ This early activity in T cell activation makes the NR4A1 promoter ideal for the expression of CAR receptors. The NR4A gene products are themselves TFs that bind to NR4A‐transcription sites (NurRE) present in the promoter regions of a set of inducible genes. Repeated NurRE sites have previously been included in an inducible promoter design for payload delivery.^31^

We hypothesized that the use of inducible promoters to express a CAR in response to antigen encounter, would result in a lowered exhaustive phenotype and increased persistence and function. To accomplish this, we compared the activity of constitutive (EF1α), synthetic inducible (6NFAT‐NFκB and 2NFAT‐2NurRE) and native early activation promoter (NR4A1) under a variety of conditions to assess both CAR expression and function pre‐ and post‐T cell activation. Here, we demonstrate the first study utilizing the NR4A1 promoter for the expression of a CAR in the context of solid tumors. Results from this study showed that the expression of the FRP5‐CAR was contingent on recognition of antigen and T cell activation, resulting in increased CAR expression from a low baseline to expression levels, which showed equivalent function to CARs expressed constitutively by EF1α. Therapeutic levels of CAR expression were attained in vivo by NR4A1‐driven CARs. This study has shown that NR4A1‐driven CARs are able to mount an effective and persistent antitumor effect in both in vitro and in vivo settings. This manuscript details the superior performance of the NR4A1 promoter as compared to existing inducible promoters. The NR4A promoter offers an additional advantage with its short length (~365 bp vs. ~1100 bp for EF‐1 promoter) thereby minimizing cargo sizes in transfer vectors.

## RESULTS

### Identification of NR4A1 as a promising candidate promoter

**Figure 1:**
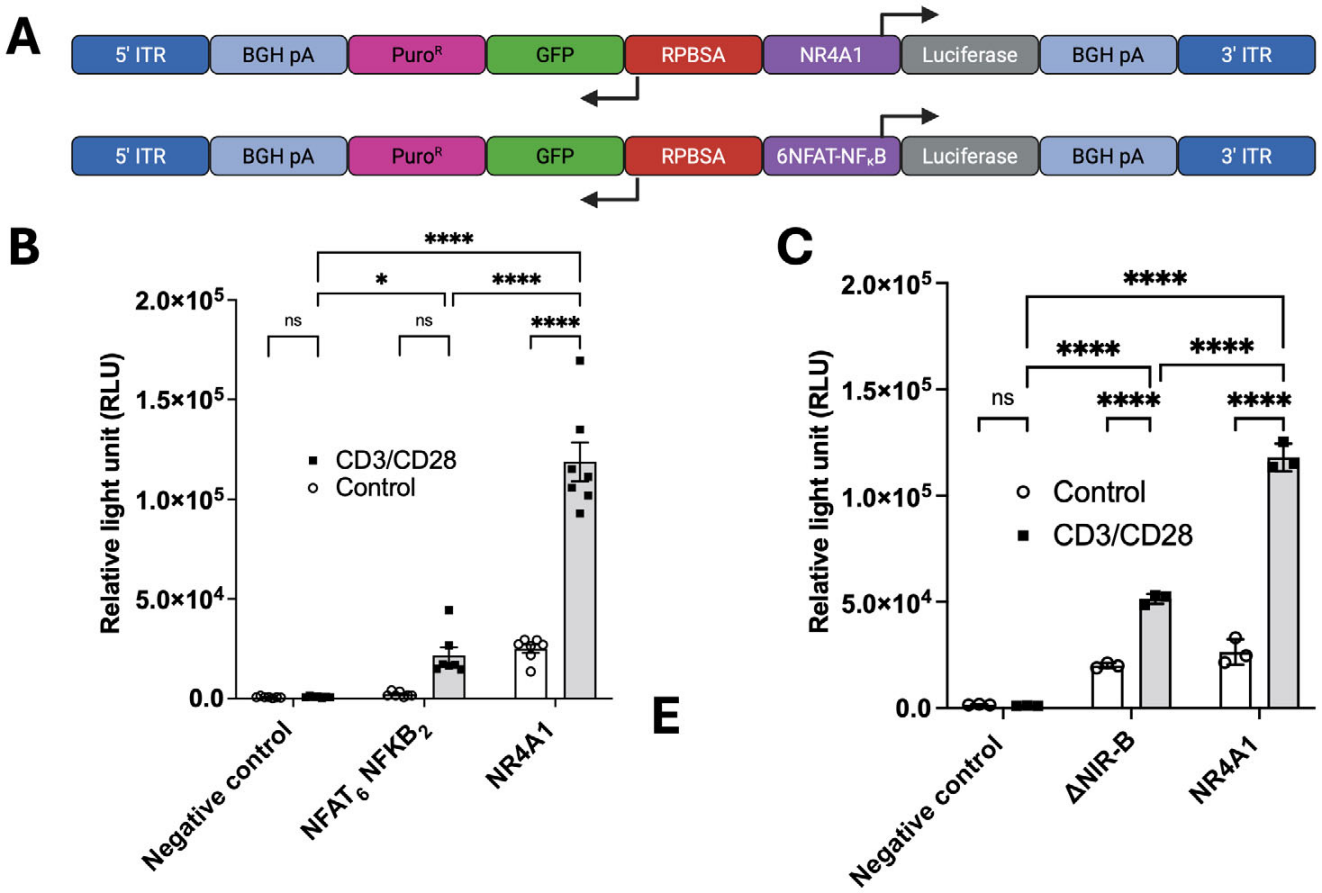
NR4A1 minimal promoter shows activation‐inducible gene expression following T cell activation. **(a)** Schematic illustration of Sleeping Beauty expression cassette bearing activation‐inducible promoter NFAT_6_NFκB_2_ and NR4A1 minimal, driving the expression of the firefly luciferase reporter gene. **(b)** Expression of luciferase in Jurkat T cells transfected with activation‐inducible promoter gene cassettes illustrated in (A), pre‐ and 24 h poststimulation with αCD3/CD28 antibodies. **(c)** Expression of luciferase in Jurkat T cells bearing wildtype NR4A1 minimal and deletion mutant (ΔNIR‐B) NR4A1 minimal promoter driving expression of luciferase pre‐ and 24 hr poststimulation with αCD3/CD28 antibodies. Significance determined by multiple‐comparison two‐way ANOVA, *n* = 6.

To assess the functionality of AI promoters in T cells, we generated transposon plasmids in which the expression of the firefly luciferase gene was placed under the control of candidate promoters. The minimal NR4A1 promoter (−315 to +46 bp) was amplified from genomic DNA of activated human PBMCs via targeted polymerase chain reaction (PCR), with a six‐repeat (four cis‐ two trans‐acting) NFAT‐NFκB (6NFAT‐NFκB) promoter used as a comparator to provide a well‐established inducible construct for comparison (Figure 1a). Jurkat T cells were transfected with the respective constructs and activated via incubation with αCD3 and αCD28 antibodies for 24 h, followed by assessment of luciferase signal. Both AI‐promoter constructs showed an increase in luciferase signal following activation when compared to an empty vector control (Figure 1b). Despite showing promising inducibility, the NR4A1 promoter construct also showed detectable luciferase expression in the absence of stimulation (Figure 1b). Previous reports have indicated that the removal of the central region of the NR4A1 promoter, known as region B, can reduce basal expression while maintaining overall expression in Leydig cells.^31^ To examine the applicability of such an approach in T cells, we removed region B from the NR4A1 promoter and compared the mutated construct (ΔNIR‐B) to the wild‐type NR4A1 (Figure 1c). Interestingly, the removal of region B from the promoter was shown to have no impact on basal luciferase signal; however, there was a reduction in overall expression levels (Figure 1c). Further development of the NR4A1 promoter for inducible gene expression was carried out with the full‐length promoter.

### AI promoters are capable of driving CAR expression in primary human T cells

**Figure 2:**
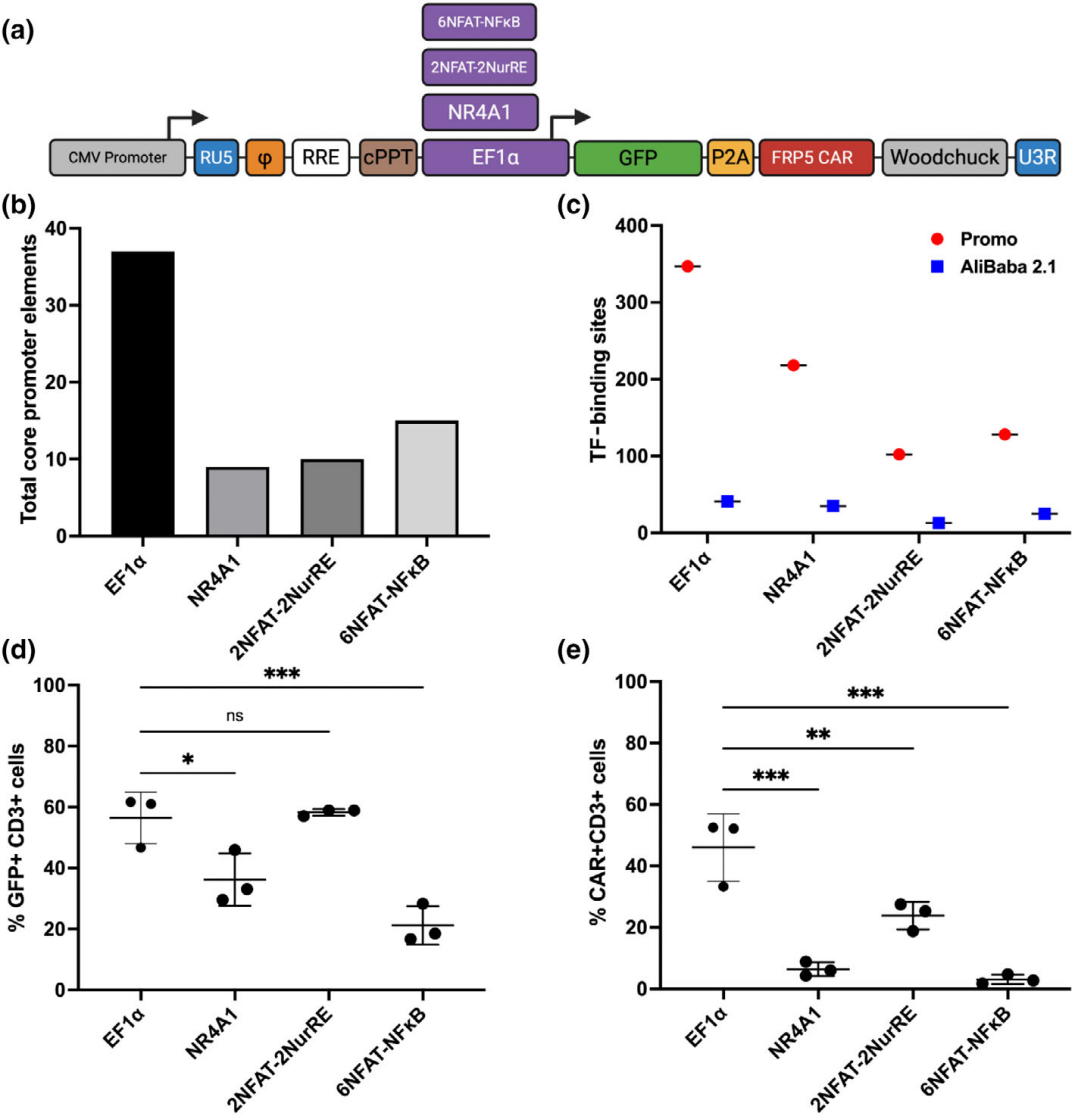
In silico comparative analysis of activation‐inducible promoter structure and composition. **(a)** Schematic illustration of third‐generation lentiviral expression cassettes containing alternative promoter constructs driving the expression of GFP‐P2A‐HER2CAR. **(b)** Total number of core promoter elements in each promoter as predicted by the YAPP software. **(c)** Total number of predicted transcription factor binding sites in each promoter construct analyzed using AliBaba2.1 and PROMO software. **(d)** Expression of GFP in primary human T cells transduced with third‐generation lentiviral vectors. **(e)** Primary human T cells were transduced at an MOI of 40 and expanded for 72 h prior to assessment via flow cytometry. Expression of HER2 CAR in primary human T cells transduced with third‐generation lentiviral vectors. Primary human T cells were transduced at an MOI of 40 and expanded for 72 h prior to assessment via flow cytometry.

To investigate the use of AI‐CARs for use in solid tumor CARs, the Her2 recognizing FRP5 scFv, tagged with cmyc and linked to second‐generation CD28 CD3ζ signaling domains was chosen for inducible expression, (Figure 2a). The constitutive promoter EF1α was selected for comparison with the AI promoters based on previous publications of the McLellan research group, which highlighted its transcript expression strength and superior function in CAR T cells.^21^ The AI promoters 6NFAT‐NFκB, 2NFAT‐2NurRE and NR4A1 were cloned in place of EF1α in otherwise identical third‐generation lentiviral pCCL.SIN vectors. The 6NFAT‐NFκB comprised a response element containing six NFAT TF recognition sequence repeats with a single repeat of the NFκB TF recognition sequence placed upstream of the minimal human IL‐2 promoter. Likewise, the 2NFAT‐2NurRE promoter was composed of two NFAT TF response elements upstream of two TF response elements recognized by NR4A that were linked to the minimal elements of the Herpes simplex virus (HSV) thymidine kinase (TK) promoter, referred to as mini‐TK. The minimal elements of the NR4A1 promoter were amplified from genomic DNA sourced from healthy donor PBMCs.

Promoters were analyzed using PROMO (version 8.3 of TRANSFAC^32^) and Alibaba (version 2.1) to compare each promoter's core elements (Figure 2b). Although RNA polymerase II binds to relatively noncanonical regions, the transcription factor II B (TFIIB) recognition element (BRE), TATA box, initiator (INR) element, motif ten element (MTE) and downstream core promoter element (DPE) are central elements of promoter composition. EF1α contained the highest number of core promoter elements followed by 6NFAT‐NFκB. Of note, the number of elements was not normalized to promoter size during this comparison. We next investigated the total number of TF‐binding sites (TFBS) found within each promoter (Figure 2c). The distribution of TFBS is plotted as a percentage of TFBS found across all four promoters. The EF1α promoter contained the highest number of TFBS, followed by NR4A1, 6NFAT‐6NFκB and 2NFAT‐2NurRE, respectively. Transduction of primary human T cells using lentiviral cassettes containing the respective promoters showed a significant increase in expression of GFP in EF1α CAR T cells compared with the NR4A1 and 6NFAT‐NFκB CARs, while no significant difference compared with the 2NFAT‐2NurRE design (Figure 2d). The expression of CAR on the surface of transduced cells indicated a significant difference between EF1α‐driven CARs and all AI‐CAR designs, highlighting the minimal baseline expression of the AI‐CARs at rest (Figure 2e). The expression of CAR was measured by staining against a c‐myc tag within the hinge region.

### Activation‐responsive promoters' express CAR cassettes in response to stimulation

**Figure 3:**
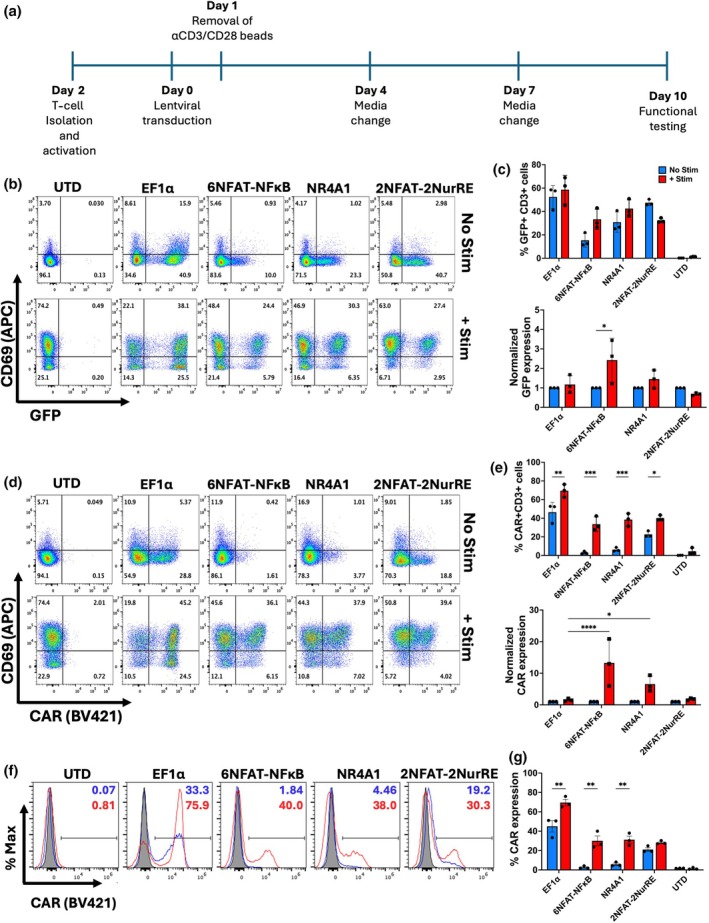
Activation‐inducible promoters produce a functional response to stimulation in primary human T cells. **(a)** Schematic illustration of the ex vivo expansion timeline for primary T cells utilized throughout this study. Cells were cultured in CTS OpTimizer T cell Expansion media supplemented with 50 IU/mL IL2. **(b)** Representative flow cytometry plots showing the expression of the CD69 activation marker and GFP both prior to stimulation (No stim) and 24 h following coincubation with αCD3/CD28 beads (+ Stim). **(c)** GFP fluorescence in CD3^+^ cells both prior to stimulation and after 24 h of incubation was expressed as either an absolute percentage of positive cells (Top) or normalized to basal expression levels (Bottom). **(d)** Representative flow cytometry plots showing the expression of the CD69 activation marker and HER2 CAR both prior to stimulation (No stim) and 24 h following coincubation with αCD3/CD28 beads (+ Stim). **(e)** CAR expression in CD3^+^ cells both prior to stimulation and after 24 h of incubation expressed as either an absolute percentage of positive cells (Top) or normalized to basal expression levels (Bottom). **(f)** Representative flow cytometry plots showing the expression of the HER2 CAR on the surface of CD3^+^ cells both prior to stimulation (−Antigen) and following 24 h of coincubation with HER‐Fc fusion protein (+ Antigen). (g) CAR expression in CD3^+^ cells both prior to stimualtion (No stim) and after 24 h of incubation with HER2‐Fc (+ Stim). Statistical significance was determined using a Two‐way ANOVA with Holm–Sidak correction for multiple comparisons (**P < 0.01, ***P < 0.005, ****P < 0.001).

Next, to assess the ability of the inducible promoters to respond to T cell activation through the CD3 and CD28 stimulatory pathway, primary T cells were transduced with either inducible or EF1α driven FRP5 CARs and cultured with anti‐CD3 and anti‐CD28 coated beads (Figure 3a). The activation of transduced primary T cells was measured by expression of surface marker CD69; additionally, GFP and surface CAR expression was measured by c‐myc tag staining.

Transduced T cells were assessed for CD69 expression prestimulation and following 24 hr incubation with stimulatory beads (Figure 3b). Transduced cells containing inducible promoters showed no significant percent increase in the GFP when compared to prestimulation (Figure 3c; top); however, there was a significant increase in the fold change of GFP within the NFAT system (Figure 3c; *lower*). There was a significant percent increase in CAR^+^ cells observed in T cells transduced with all expression systems poststimulation when compared to cells at rest (Figure 3d, e). Interestingly, there was a significant increase observed in the expression of EF1α‐driven CAR poststimulation, highlighting potential intrinsic upregulation of genes under EF1α control. However, when normalized to CAR transduction efficiency, only NFAT and NR4A1, and not the NFAT‐2NurRE promoter, showed a significant increase in CAR expression.

In addition to CD3 and CD28 stimulation, inducible promoters were assessed for their ability to increase surface CAR expression following exposure to Her2 antigen. Transduced T cells were cultured in the presence or absence of plate‐bound Her2 antigen for 24 h. Following antigen incubation, transduced T cells were assessed for CAR surface expression by flow cytometry (Figure 3f, g). A significant increase in percent CAR expression was observed in the EF1α, 6NFAT‐NFκB and NR4A1‐driven cassettes, consistent with the CD3/28 stimulation assay, while the 2NFAT‐2NurRE did not show a significant increase in CAR expression following antigen exposure. These results were in line with those seen when CAR T cells were stimulated with αCD3/CD28 antibodies.

### Self‐driving CAR cassettes are functionally comparable to constitutive EF1α CAR

**Figure 4:**
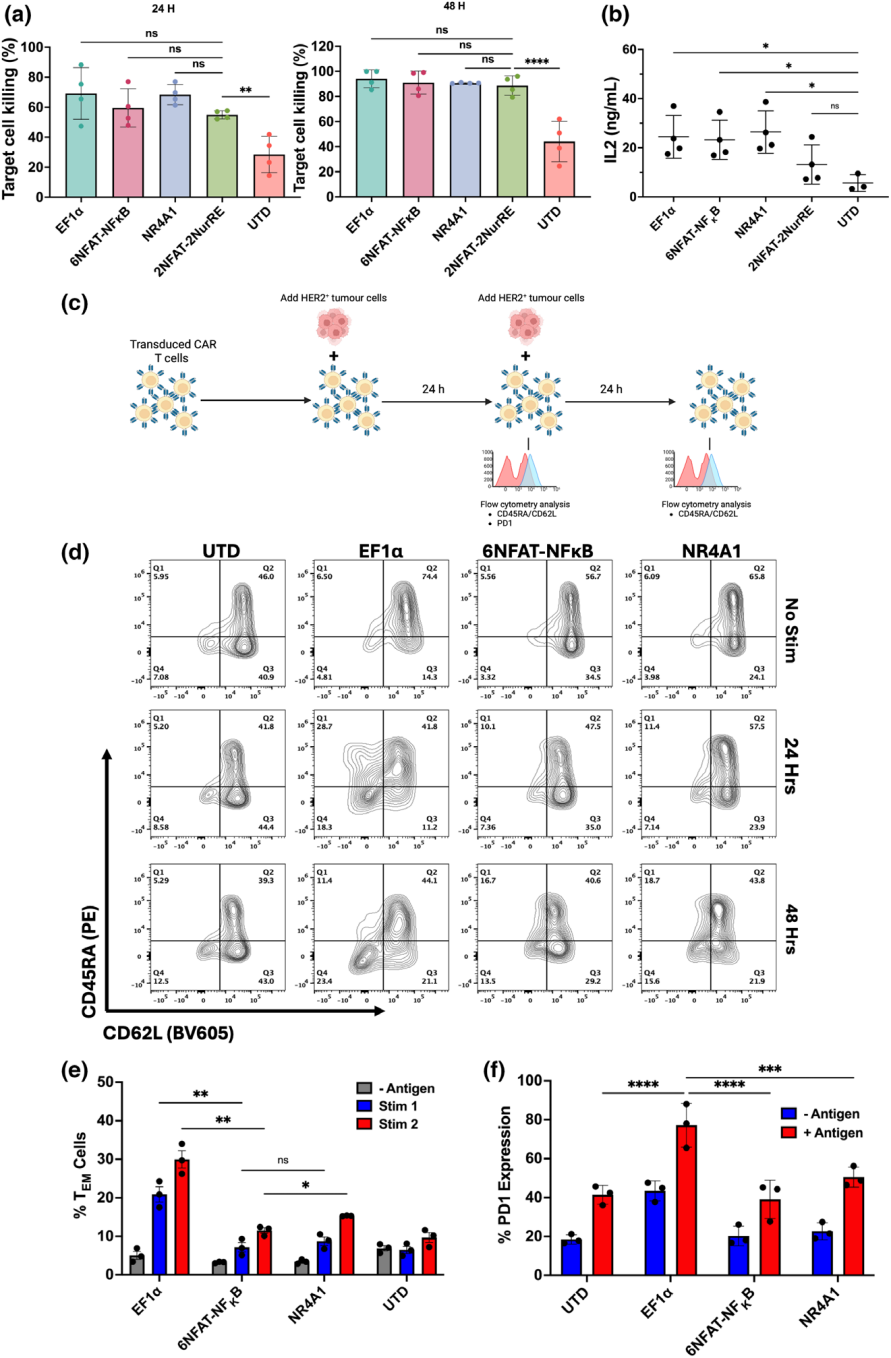
Activation‐inducible promoters show responsiveness to stimulation in primary human T cells. **(a)** Cytotoxicity assay showing specific lysis of HER2^+^ target cells by EF1α‐ and AI‐CAR T cells. T cells transduced with either EF1α‐ or AI‐CAR bearing lentivirus were incubated with target cells at a 2.5:1 E:T ratio for either 24 or 48 h prior to assessment of luciferase signal. **(b)** Secretion of IL2 by T cells transduced with either EF1α‐ or AI‐CAR bearing lentivirus and untransduced controls following incubation with HER2^+^ target cells. Each data point represents an independent healthy donor (*n* = 4). Plots represent mean ± SEM. **(c)** Schematic illustration depicting the timeline of assay utilized to measure the behavior of AI‐CAR T cells following incubation with HER2^+^ target cells. CAR T cells were incubated with tumor cells at a 2.5:1 E:T ratio for 24 h. The cells were either removed for flow‐cytometric analysis or supplemented with additional tumor cells for a further 48 h. **(d)** Representative flow cytometry plots showing the expression of CD45RA and CD62L following one (24 h) or two (48 h) incubations with MCF7‐HER2‐Luc target cells. Flow plots are representative of three independent experiments and healthy donors (*n* = 3). **(e)** Pooled results showing the relative percentage of CD3^+^ T cells in the T_EM_ compartment (CD45RA/CD62L) during antigenic stimulation. Bars represent mean ± SEM for three independent healthy donors (*n* = 3). **(f)** Expression of PD1 by CAR T cells following incubation with HER2+ target cells. T cells were coincubated for 24 h prior to flow‐cytometric analysis. Bars represent mean ± SEM for three independent healthy donors (*n* = 3). Statistical significance was determined using a Two‐way ANOVA with Holm–Sidak correction for multiple comparisons (**P < 0.01, ***P < 0.005, ****P < 0.001).

Following confirmation that 6NFAT‐NFκB and NR4A1 drive enough basal CAR expression for effective antigen recognition, we then sought to assess the capacity of 6NFAT‐NFκB, 2NFAT‐2NurRE and NR4A1‐driven CAR to mediate cytotoxic killing, cytokine production and T cell expansion. EF1α‐driven FRP5 CAR was used as a control for constitutive CAR T cell function. MCF7 cells were transfected with the full‐length Her2 antigen to ensure stable antigen expression. Transduced T cells were cocultured with MCF7‐Her2^+^‐luciferase cells independently for 24 and 48 h at a 2.5:1 effector to target ratio (Figure 4a). Both constitutive and inducible promoter‐driven CAR cassettes showed a significant increase in target cell killing compared with empty vector and untransduced control T cells. There was no significant difference in cytotoxic capacity between EF1α and 6NFAT‐NFκB, 2NFAT‐2NR4A and NR4A1‐driven CAR cassettes at either timepoint. In addition, the functionality of CAR T cells transduced with inducible promoters was assessed for release of IL‐2 (Figure 4b) where secretion of IL‐2 following coincubation with target cells was observed in all CAR T cells with the exception of the NR4‐NFAT system. Having shown reduced IL‐2 secretion compared with other CAR T cells, and coupled with the previous finding of an unfavorable gene induction pattern, the 2NFAT‐NurRE promoter was excluded from downstream analysis.

**Figure 5:**
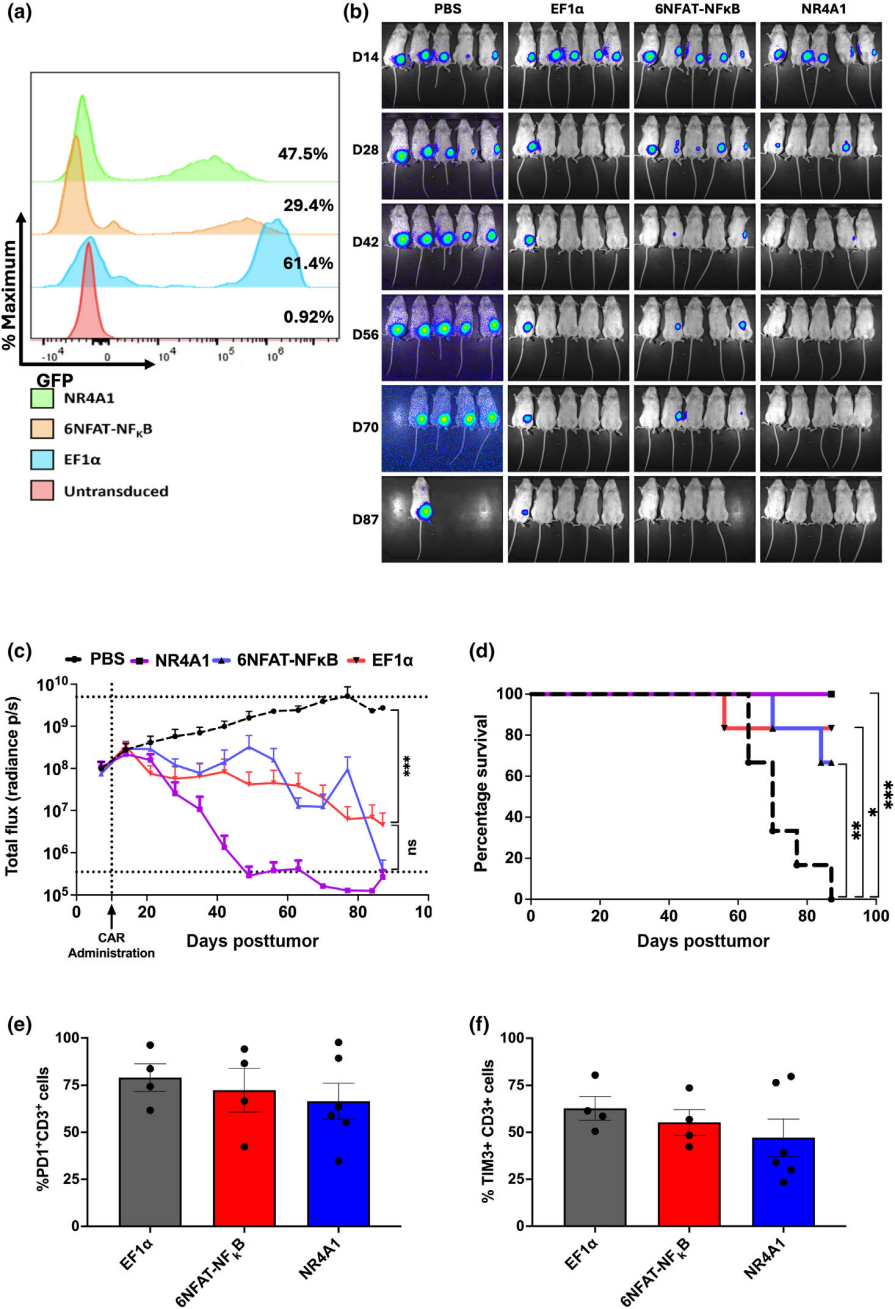
AI‐CAR T cells show comparable in vivo efficacy to EF1α CAR T cells. **(a)** Representative flow cytometry plots showing the expression of GFP in CAR T cells prior to intravenous injection. CAR T cells were incubated with αCD3/CD28 antibodies for 24 h prior to the assessment of GFP expression to determine transduction rates via flow cytometry. **(b)** Tumor burden in NSG mice based on luciferase expression of MCF7‐HER2^+‐^luciferase tumors at various timepoints throughout the study (*n* = 6 mice per group; a batch of 5 imaged mice is shown). **(c)** Grouped radiance values for tumors from each treatment group are displayed as a function of time. Data points and error bars represent mean + SEM. Statistical significance was calculated by two‐way ANOVA with Holm–Sidak correction for multiple comparisons (*N* = 6 mice per group). **(d)** Survival curves for mice treated with PBS vector control or CAR T cells post‐tumor administration. Statistical significance was determined by log‐rank Mantel–Cox test. ***P < 0.001 (*N* = 6 mice per group). **(e)** The expression of PD1 on T cells isolated from NSG mouse spleens upon removal from the study was measured via flow cytometry. **(f)** The expression of TIM3 on T cells isolated from NSG mouse spleens upon removal from the study was measured via flow cytometry. Each datapoint represents a single mouse, with bars representing mean ± SEM. (*n* = 3, 4, 6 for EF1α, 6NFAT‐NFκB and NR4A1, respectively).

To probe the potential impact of reduced basal CAR expression on CAR T cell fitness, we also measured the expression of activation markers (CD69) and T cell phenotype (CD45RA and CD62L) during ex vivo expansion. We found that the promoter driving CAR expression had little impact upon the level of activation marker expression in the absence of antigenic stimulation, indicating that the level of CAR expression seen in EF1α CAR T cells was not inducing significant tonic signaling (Supplementary figure 4A). The same was true for T cell phenotype, with all constructs maintaining a primarily central memory‐like (CD45RA^‐^/CD62L^+^) phenotype throughout the entirety of *ex vivo* expansion (Supplementary figure 4B). Finally, we assessed the behavior of different promoter‐driven CAR T cells following repeated antigen encounter through the measurement of T cell phenotype and exhaustion marker expression (Figure 4c, Figure 5). Transduced T cells were coincubated with target cells for 24 h, after which cells were either removed for analysis or replenished with fresh tumor cells and coincubated for a further 24 h. As expected, all CAR T cells showed a shift toward an effector memory phenotype (CD45RA^‐^/CD62L^‐^) following antigen encounter (Figure 4d, Supplementary figure 5). Interestingly, this shift was seen to be far more pronounced in the EF1α CAR T cells, while CAR T cells with AI promoters showed a less differentiated phenotype at both 24 and 48 h (Figure 4d, e). The expression of PD‐1 was significantly upregulated in antigen‐stimulated EF1α CAR T cells compared with 6NFAT‐NFκB‐ and NR4A1‐driven AI‐CARs CAR T cells.

Following the findings that 6NFAT‐NFκB‐ and NR4A1‐driven AI‐CARs were comparable in effector function but showed a reduction in exhaustion marker expression, further validation was sought through *in vivo* models.

### Self‐driving CARs show high efficacy in vivo tumor models

To further determine the therapeutic potential of the 6NFAT‐NFκB‐ and NR4A1‐driven AI‐CARs, we performed an *in vivo* study using NOD.Cg‐PrkdcscidIl2rgtm1Wjl/Szj (NSG) mice subcutaneously implanted with MCF7 Her2^+^ luciferase cells. Three treatment groups were initiated consisting of EF1α, 6NFAT‐NFκB and NR4A1 driven FRP5 CD28 CD3ζ driven CARs with an additional untreated control group. T cells were transduced with expression cassettes, expanded for 10 days and measured for transduction efficiency by GFP expression, (Figure 5b), prior to intravenous administration of 5 × 10^6^ total T cells without normalization for CAR expression. Mice were observed for 90 days, with tumor bioluminescence measured every 7 days to monitor tumor progression (Figure 5a).

The time course of tumor bioluminescence showed complete tumor clearance in the 6NFAT‐NFκB and NR4A1 treatment groups (Figure 5c). Mice within the untreated group reached the clinical endpoint first (Figure 5d), while the EF1α driven CAR treatment group showed an increase in median survival time, with one mouse lost to graft‐vs‐host disease (GVHD) and four of six mice showing complete tumor clearance. The 6NFAT‐NFκB and NR4A1 treatment groups showed an increase in the median survival time of mice, with no mice lost from the NR4A1 group and two lost from the 6NFAT‐NFκB group (Figure 5e). Complete tumor clearance was observed in both the 6NFAT‐NFκB and NR4A1 treatment groups, with complete tumor clearance seen earliest in the NR4A1 treatment group after 49 days (Figure 5e). In contrast to *in vitro* results where 6NFAT‐NFκB showed more robust CAR expression compared with the NR4A1 promoter, the in vivo results showed that the NR4A1 driven CAR showed robust and rapid clearance of tumor, while the tumor clearance took substantially longer in the 6NFAT‐NFκB group (Supplementary figure 6).

At the end of the 90‐day study, splenic T cells were isolated for phenotypic analysis. CD3^+^GFP^+^ T cells were isolated from all treatment groups. Isolated CAR T cells showed similar levels of T_EFF_, T_CM_, T_EMRA_ and T_ScM_ (Supplementary figure 7), although there was a slight increase in the percent of T_EMRA_ and T_SCM_ in the inducible CARs compared with EF1α. Analysis of exhaustion markers showed no significant increase in PD‐1 or TIM‐3; however, consistent with in vitro results, NR4A1 had the lowest expression of both exhaustion markers compared with EF1α or 6NFAT‐NFκB driven CARs, (Figure 5f), in vivo studies were repeated and showed consistent findings (Supplementary figure 8). These results show that CAR T cells in which the expression of the CAR is driven by an AI promoter are capable of inducing robust antitumor responses in NSG mice comparable to that of EF1α CAR T cells.

## DISCUSSION

This study is the first to use the NR4A1 promoter to express a 2nd generation CAR cassette, though recent publication by Chen et al. has highlighted the use of the NR4A2 promoter for the controlled expression of cytokine payload delivery.^31^ While the self‐driving CAR driven by 6NFAT‐NFκB showed the most promising results in vitro, the minimal NR4A1 promoter had the highest efficacy in vivo. NR4A1‐driven FRP5 CAR expression was robustly induced upon T cell stimulation and showed comparable in‐vitro performance to the constitutive EF1α‐driven CAR, while also achieving complete tumour clearance in vivo.

The CAR expression driven by AI promoters 6NFAT‐NFκB and NR4A1 exhibited minimal background expression and upregulated CAR expression following T cell activation and antigen encounter. Significant CAR induction was observed from both the 6NFAT‐NFκB and NR4A1 promoters after stimulation, whereas the 2NFAT‐2NurRE promoter displayed high background CAR expression at rest with minimal increase following T cell activation. The 6NFAT‐NFκB and NR4A1‐driven CAR demonstrated comparable cytolytic function to the constitutive EF1α‐driven CARs and exhibited similar cytokine expression levels.

Throughout this study, the accurate quantification of the relative proportion of CAR^+^ cells was made difficult due to the inducible nature of the AI‐promoter expression. While the same MOI of lentiviral vectors was maintained throughout the study, the expression of both the GFP reporter gene and FRP5‐CAR was seen to be higher in EF1α CAR T cells than in any AI‐CAR T cells (Figure 3). This is in line with the reported strength of the respective promoter constructs in the absence of antigenic stimulation.^21^, ^33^ Despite the reduced CAR expression, it was seen that AI‐CAR T cells maintained the ability to effectively lyse target cells during in vitro assays (Figure 4). This may have been in part due to the uniform high‐level expression of the target antigen on the surface of the target cells used in this study. Future work should focus on the utility of AI‐CAR T cell constructs in tumor models expressing either low or heterogeneous levels of target antigen.

Following sequential antigen stimulation, EF1α‐driven CAR T cells showed rapid polarization toward effector memory phenotypes, in contrast to AI‐CARs, which retained a higher proportion of naïve memory phenotype. Several groups have observed the rapid establishment of effector memory phenotypes which have been associated with a predisposition to increased exhaustion marker expression and reduced persistence in vivo.^26^, ^34^, ^35^, ^36^ Results shown here align with this observation in that EF1α‐driven CAR T cells exhibited increased effector memory phenotypes and a significant upregulation of PD‐1 expression.

A major challenge facing the field of CAR T cell therapy is the ability to engineer complex therapeutic products with limited DNA cargo space afforded by traditional lentiviral vectors. Several engineering strategies that accommodate large transgene payloads have been described, but they involve complex and costly workflows and are, at present, limited to ex vivo CAR T cell production.^37^, ^38^, ^39^ The NR4A1 promoter described in this manuscript is more compact than the full‐length EF1α promoter (365 bp vs. 1193 bp) but maintains the ability to drive expression of a bicistronic expression cassette, including the FRP5 CAR. The use of compact promoters in place of larger traditional promoters could allow researchers to increase the number of genetic modifications made to a given CAR T cell, allowing for complex hurdles in the field, such as the TME to be better addressed.

In addition to the compact size, the NR4A1 promoter showed the ability to effectively drive gene expression in an activation‐dependent manner. Prior investigations by Sadelain *et al*. involving the targeted insertion of CAR coding sequences under the endogenous TRAC locus, responsible for native TCR expression, resulted in lower tonic signaling, reduction in T cell differentiation and an increased rate of tumor rejection.^37^ This work highlighted the potential advantage of regulatable CAR expression in optimizing therapeutic potential compared with constitutive‐expressing alternatives. The NR4A1 construct described in this work does not place the CAR coding sequence under the control of an endogenous promoter at a predetermined genomic locus; however, it provides a lentivirus‐compatible alternative to provide context‐dependent CAR expression.

Where EF1α driven CARs showed successive polarization toward effector T cells following repeated stimulation, CARs driven by 6NFAT‐NFκB and NR4A1 promoters showed a reduced differentiation toward effector memory phenotypes and retained a higher overall proportion of naïve memory phenotype compared with EF1α driven CARs. Additionally, there was a significantly lower expression of exhaustive markers from CAR T cells driven by 6NFAT‐NFκB, which showed the lowest overall expression of PD‐1 and NR4A1, and the lowest expression of TIM‐3. Naïve memory T cells have been shown to preferentially differentiate toward central memory phenotype, which in turn has been shown to have increased persistence in vivo and is a preferred phenotype for infusion in clinical settings.^40^

Bioluminescent imaging of tumors in vivo highlighted the kinetics of tumor regression between treatment groups. While both 6NFAT‐NFκB and NR4A1‐driven CAR T cells showed tumor regression beginning around the same period, NR4A1‐driven CARs indicated a greater tumor reduction, with complete tumor clearance observed in three of six mice by Day 28. In contrast, all mice retained tumor burden within the 6NFAT‐NFκB treatment group. All mice of the NR4A1 treatment group showed complete tumor clearance by Day 70 and were sustained for the remainder of the trial.

The NR4A1‐driven CARs were able to effectively eliminate tumors in vivo, to the same efficacy as constitutively EF1α‐driven CARs in the breast‐cancer xenograft model. While no significant difference was observed within this trial between EF1α and NR4A1 treatment groups, future *in vivo* studies may show that NR4A1‐driven CARs are able to discriminate between low‐ and high‐density target antigens, improving treatment safety by reducing the prevalence of “On‐target Off‐tumor” reactions. A limitation of the NSG model employed is the development of GVHD around 50–60 days preventing prolonged monitoring of CAR T cell activity or tumor rechallenge.

The expression of the NR4A family members by T cells has been linked to exhaustion in both CD8 and CD4 populations; in particular, recent studies have observed a connection between NR4A expression and CD8 T cell fate determination.^41^, ^42^ NR4A1 and NR4A3 have been shown to compete with the bZIP TF family, including Jun, AP‐1, Fos and Bach2, while also inhibiting bZIP DNA binding motifs involved in promoting effector functions and cytokine expression.^42^ NR4A1 and NR4A3 are also responsible for increasing the expression of the FoxP3 transcription factor, leading to subsequent regualtory T cell (T_reg_) development.^43^ Additionally, NR4A1 directly regulates the expression of IRAF4 by binding to the promoter region. IRAF4 plays a significant role in the expansion and effector function of CD8 T cells, with IRAF4 deficiency linked to T cell apoptosis. The absence of NR4A1 in knockout mice resulted in elevated levels of IRAF4, which was associated with increased cytokine production and enhanced proliferation following antigen stimulation.^44^ The integration of additional NR4A1 promoter elements within CAR T cells may have sequestered transcriptional factors from endogenous NR4A promoters in a squelching mechanism.^45^ Introduction of multiple copies of exhaustion‐related TF‐binding sites (such as those contained within the NR4A‐promoter sequence) into the genome of T cells could lead to reduced availability of NR4A transcription factors, or NR4A‐associated cofactors. This in turn could potentially decrease the exhaustion of NR4A1‐driven CAR T cells occurring via other loci. However, there is currently no evidence that squelching influences the behavior of T cells with transduced promoters. While not within the scope of this investigation, future studies may seek to explore the transcriptional profile of NR4A1‐driven CARs to confirm this.

TCR triggering via calcium pathways is the primary stimulus for induction of NR4A1 in T cells.^30^ It is possible that other inflammatory cues could lead to NR4A1 activation; however, evidence for a role of cytokines or the contribution of calcium‐flux independent signaling for NR4A promoter regulation remains poorly supported by existing evidence. In addition, Guo *et al*. demonstrated that AI promoters were only minimally induced by inflammatory cytokine cocktails released by TCR‐stimulated T cells.^12^ In our study AI‐CAR T cells were exposed to IL‐2 during manufacture but were not exposed to other cytokines that may be present during CRS, such as IL‐1, IL‐6 or IL‐8. Future work may seek to interrogate the impact of various inflammatory cues on both the basal and inducible gene expression patterns of AI promoters.

Overall, this study showed the potential of the NR4A1 promoter for use in the expression of CARs, resulting in comparable cytotoxic capabilities to high‐strength constitutive promoter EF1α, while reducing the cassette size and T cell exhaustion. Fine‐tuning CAR density through AI promoters may provide a viable clinical option to improve the long‐term in vivo efficacy of CAR T cell therapy.

## METHODS

### Vector design and cloning

The minimal NR4A1 promoter −315 to +46 bp (365‐bp fragment) was amplified from genomic DNA of activated human PBMCs via targeted PCR and cloned within the Sleeping Beauty plasmid vector upstream of the firefly luciferase gene using restriction sites SalI and KpnI.

AI promoters, minimal NR4A1, 6NFAT‐NFκB and 2NFAT‐2NurRE were cloned into the pCCLsin lentiviral transfer vector via Gibson homology cloning. The pCCLsin lentiviral transfer vector encoded a bicistronic expression cassette consisting of GFP‐P2A‐FRP5 CAR under the control of the EF1α promoter. The FRP5 CAR used in this study consisted of the FRP5 scFv, c‐myc epitope tag, CD8 hinge, CD28 transmembrane domain, CD28 costimulatory domain and CD3ζ activation domain. The pCCLsin:EF1α‐GFP‐FRP5 CAR plasmid was digested with EcoRV and XbaI to remove the EF1α promoter, with the three respective AI promoters PCR amplified with primers sharing homology to the digested pCCLsin backbone. All primers are outlined in Supplementary table 1.

### Flow cytometry

Spectral cytometry was carried out using the Cytek Aurora. All data analysis was carried out using the FlowJo™ V10. As a general staining protocol, cells were washed with flow buffer and incubated with 50 μL of primary antibody on ice for 30 min. Following staining, cells were washed twice and stained with secondary reagent where applicable. Cells were fixed using a cell fixation buffer prior to acquisition. All antibodies used in this study are listed in Supplementary table 2.

### Transfection of target cell lines

The MCF‐7 cell line was maintained in Dulbecco's modified Eagle medium (catalog number: 12 100–046; Gibco) supplemented with 10 percent FCS using ATCC guidelines for cell density. Prior to transfection MCF‐7 cells were seeded at 3 × 10^5^ in a 6‐well plate at a total volume of 4 mL. MCF‐7 cells were transfected with Sleeping Beauty vectors using Lipofectamine 3000 reagent according to manufacturers' instructions. Cells were transfected with target vectors at a ratio of 5:1 transfer:transposase. Transfected cells were selected using appropriate antibiotics based on vector‐conferred resistance.

### Lentiviral production

Lentiviral particles were generated in HEK293T (ATCC CRL‐3216) producer cells using lipofectamine 3000 (L300075; Thermo Fisher) transfection, according to the manufacturers protocol. The day prior to transfection, 2.2 × 10^7^ HEK293T cells were seeded in T175 flasks in high glucose Dulbecco's modified Eagle medium (catalog number: 12100‐046; Gibco) supplemented with 10 percent FCS and incubated for 18 h at 37°C with 5 percent CO_2_ to allow adherence. HEK293T cells were transfected with 13.8 μg of each of the packaging vectors (pRSV‐Rev, pMDLg/pRRE, pCMV‐VSV‐G) alongside 13.8 μg of the transfer vector (pCCLSin; kindly gifted by Luigi Naldini, Fondazione Telethon, Milan, Italy). 18 h following transfection, culture supernatant was replaced with 32 mL of OptiMEM (catalog number: 31985088; Thermo Fisher) supplemented with 5 percent FCS and incubated for a further 24 h at 37°C with 5 percent CO_2_. Culture supernatant was centrifuged at 2000 × *g* for 20 min to remove cell debris, followed by ultracentrifugation at 120 000 × *g* for 2 h 30 min to concentrate viral particles. Viral pellets were resuspended in T cell media and stored at −80°C.

Functional viral titer was determined by transduction of HEK293T cells. A range of viral dilutions were made using Dulbecco's modified Eagle medium supplemented with 10 percent FCS and 8 μg/mL of polybrene (catalog number: H9268‐5G; Sigma‐Aldrich), 500 μL of viral dilutions were added to HEK293T cells seeded at 5 × 10^4^ and spin‐inoculated by centrifugation at 450 × g for 20 min. Transduced HEK293T cells were incubated for 16 h, and media was replaced with Dulbecco's modified Eagle medium supplemented with 10 percent FCS and further incubated for 72 h. Frequency of reporter gene expression was determined by spectral flow cytometry, and viral transducing units mL^−1^ were calculated using the formula:

$$\text{Transducing Units}/\mathrm{mL}\;\left(\mathrm{TU}/\mathrm{mL}\right)=\text{Frequency of}\;\mathrm{GFP}\;\times \;\left[50,000/\text{volume}\right]\;\times \;\text{Dilution factor}$$

### Primary T cell transduction and culture

Human PBMCs were isolated from healthy donors who had provided informed written consent as part of protocol H18/089, which was approved by the University of Otago Human Ethics Committee with written consent (ethics approval number: H18‐089). Human T cells were isolated from cryopreserved PBMCs using the EasySep™ T cell Isolation Kit (no.: 17951; STEMCELL) as per the manufacturers protocol. Briefly, cryopreserved PBMCs were thawed in prewarmed TCE and rested overnight. The following morning cells were washed and resuspended in T cell isolation buffer at a concentration of 5 × 10^7^ cells/mL in a polystyrene round bottom tube (no.: 352008; In Vitro Technologies). T cell isolation cocktail was added at 50 μL/mL and gently mixed, followed by incubation for 5 min at RT. During incubation, RapidSheres™ were well mixed and then added to the incubated mixture. The PBMC mixture was then topped up to 2.5 mL total volume with T cell isolation buffer and incubated in the EasySep™ (no.: 18000; STEMCELL) magnet for 3 min. Following incubation, cells were transferred to a fresh polystyrene tube by inversion, ensuring one continuous motion to minimize non‐T cell contamination. The isolated T cells were counted and resuspended in TCE media with 50 U/mL IL‐2 (no.: 200–02‐100 μg; PeproTech) at a concentration of 1 × 10^6^ cells/mL. Isolated T cells were activated using Dynabeads Human T‐Activator CD3/CD28 (ThermoFisher #A35684) for two days prior to transduction. T cells were cultured at 3‐day intervals with fresh IL‐2 supplemented media. At 24 h prior to T cell transduction, 24‐well plates were coated with 300 μL of 40 μg ml^−1^ retronectin (catalog number: T100A/B; TAKARA) for 18 h at 4°C. Retronectin was removed, and wells were blocked with 2 percent FCS/PBS buffer for 15 min at room temperature, before addition of lentiviral particles at a 40:1 MOI to the plate. The volume was adjusted to 500 μL before centrifugation at 2000 × g for 2 h 30 min at room temperature. 48 h after T cell activation, T cells were added to virus‐coated wells and spin‐inoculated at 450 × g for 20 min, then incubated at 37°C with 5 percent CO_2_. 18 h after transduction, DynaBeads were removed from T cell culture and T cells passaged in fresh medium supplemented with 50 U/mL and repassaged every 2 or 3 days at a density of 1 × 10^6^ cells mL^−1^.

### Activation assays

Activation assays were performed to test the inducibility of AI‐promoter constructs using either αCD3/CD28 antibodies or through plate‐bound HER‐2 protein antigen. For antibody‐based screening, the base of a 96‐well plate was coated with 2.5 μg/mL (100 μL/well) αCD3 antibody (no.: 317326; Biolegend) diluted in PBS overnight at 4°C. The following day, T cells were counted and resuspended at a concentration of 1 × 10^6^ cells/mL in prewarmed TCE supplemented with 50 U/mL IL‐2 and 5 μg/mL αCD28 antibody (no.: 302923; Biolegend). A total of 100 μL of cell suspension was added to each well, and plates were incubated at 37°C with 5 percent CO_2_ for 24 h. Cells were then removed from wells, washed with flow wash buffer and stained for analysis via flow cytometry.

For protein‐based antigen, the base of a 96‐well plate was coated with 2 μg/mL of HER2‐Fc fusion protein (100 μL/well) diluted in PBS overnight at 4°C. The following day, T cells were counted and resuspended at a concentration of 1 × 10^6^ cells/mL in prewarmed TCE supplemented with 50 U/mL IL‐2. A total of 100 μL of cell suspension was added to each well, and plates were incubated at 37°C with 5 percent CO_2_ for 24 h. Cells were then removed from wells, washed with flow wash buffer and stained for analysis via flow cytometry.

### Cytotoxicity assays

Primary T cells transduced with their respective CAR constructs were screened for their cytotoxic activity against target cells using a luciferase‐based cytotoxicity assay. The day prior to testing, the wells of a 96‐well plate were coated with MCF7‐HER2‐Luciferase (MCF7‐HER2‐Luc), MCF7‐Luc or HEK293T‐Luc target cells at a density of 5 × 10^4^ cells/well. Plates were maintained overnight at 37°C with 5 percent CO_2_. The following day, T cells transduced with either EF1α‐ or AI‐CAR bearing lentivirus were cultured and resuspended at the appropriate concentration based on the desired effector: target (E:T) ratio, with a total of 100 μL of cells added to each well. The cells were cocultured for either 24 or 48 h at 37°C with 5 percent CO_2_. Following coincubation, 50 μL of cell culture medium was removed and replaced with an equal volume of firefly luciferase one‐step glow assay working solution (no.: A1048501; Pierce). Plates were mixed by shaking and incubated for 45 min in the dark at RT. Following incubation, 50 μL of media from each well was transferred to a flat‐bottom black 96‐well plate (no.: 396751; Interlab) and luminescence was measured at 540/585 nm using a Varioskan LUX plate reader. The percentage of target cell killing was determined using the following equation:

$$\text{percent Killing}=\left[\left(\text{TargeT cells only}\right)-\left(\text{Effector}+\text{TargeT cells}\right)\right]/\text{TargeT cells only}$$

### Cytokine detection ELISA

The expression of IL‐2 and IFNγ was measured via sandwich ELISA. Nunc 96‐well flat‐bottom maxisorp plates (#44‐2404‐21; Thermo Fisher) were coated with 100 μL of either IFNγ or IL‐2 capture antibody (nos.: 551221 and 55051, respectively; BD Bioscience) diluted in PBS (2 μg/mL) overnight at 4°C. Coated plates were washed three times with 300 μL/well 0.05 percent Tween20/PBS before blocking with 200 μL of 1 percent FCS/PBS for 15 min at RT. The blocking reagent was removed by flicking, and 100 μL of cell supernatant was loaded into the wells. Alongside samples, protein standards of either recombinant IL‐2 or IFNγ (no.: 300‐02‐100 μg; Peprotech) diluted in 1 percent FCS/PBS were serially diluted in duplicate from 40 ng/mL to 0.078125 ng/mL to generate a standard curve. All subsequent incubations were carried out at 37°C for 1 h. Plates were washed as previously described, and 100 μL of biotinylated antibody specific for either IL‐2 (no.: 555040; BD Pharmingen) or IFNγ (no.: 5514550; BD Pharmingen) was added to the wells, followed by subsequent incubation. Plates were then washed and 100 μL of horse radish peroxidase‐conjugated streptavidin (no.: 11089153001; Sigma‐Aldrich) diluted 1/5000 in 1 percent FCS/PBS was added to each well, followed by a further incubation step. Incubated plates were washed again before 50 μL of 3,3′,5,5′‐tetramethybenzidine (TMB, no.: 00–202‐3; Thermo Fisher Scientific) was added to each well to allow for color to develop. Once sufficient reaction development was observed, a 25 μL volume of 2 N H2SO4 was added to stop the reaction. ELISA plates were imaged with the Varioskan LUX, and absorbance at 450 nm was measured.

### Serial stimulation assay

The wells of a flat‐bottom 96‐well plate were seeded with 1 × 10^5^ MCF7‐HER2‐Luc target cells and incubated overnight at 37°C with 5 percent CO2. The following day, T cells were cultured and resuspended at a concentration of 1 × 10^6^ cells/mL in fresh TCE supplemented with IL‐2. A total of 100 μL of resuspended T cells was added to each preseeded well, in duplicate, and coincubated for 24 h. Following coincubation, one well for each construct was resuspended, washed and stained for analysis by flow cytometry. Alternatively, the remaining wells were removed, washed and repassaged onto fresh target cells for a further 24 h of incubation. At 48 h postincubation, these cells were resuspended, washed and stained for analysis by flow cytometry.

### Animal studies

NOD‐scid IL2R gamma^null^ (NSG) mice were obtained from Jackson Laboratory (Bar Harbor, ME) and bred under specific pathogen‐free conditions at the University of Otago Animal Research Center. The Animal Ethics Committee (AEC) from the University of Otago Animal Research Center approved the animal studies under the Animal Use protocols 19–146.

Age matched female NSG mice (*n* = 6 per treatment) were injected in the ventral lateral flank with 2 × 10^6^ MCF7‐Her2‐Luciderase cells via subcutaneous injection following standard operating procedure (SOP) (SOP No.: AWO 002), University of Otago Animal Welfare Office. Seven days post‐tumor administration, mice were imaged to assess tumor engraftment and allocated to treatment groups based on tumor luminescence so that all groups had similar tumor burden. Treatment groups consisted of PBS, or 5 × 10^6^ T cells isolated from healthy donor PBMCs transduced with EF1α‐Her2 CAR T cells, NR4A1‐Her2 CAR or 6NFAT_NFκB‐Her2 CAR. Treatments were administered by intravenous injection of the lateral tail vein according to SOP No.: AWO 004.

Tumor burden was measured by digital caliper measurement every second day following tumor administration, volume calculated by: V = 0.5 × length × width.^2^ Tumor luminescence was measured every 7 days using the Perkin Elmer IVIS X5 system. Mice were administered 150 mg/kg of D‐luciferin potassium salt (GoldBio: LUCK‐1G) by intraperitoneal injection (SOP No.: AWO 003). Luciferin was incubated for 6 min, then mice were anesthetized by isoflurane induction until effect. Luciferin was further incubated until 12 min total postadministration, after which mice were positioned within the system and imaged at 1‐min intervals for a total of 5 min. This procedure was performed weekly until the end of study.

Tumor luminescence was analyzed using Living Image software, version 4.1 (Perkin Elmer). The bioluminescence signal flux for each mouse was expressed as total flux of photons/sec. Results are expressed as mean ± standard deviation (SD) of the total flux of photons/sec of three to six mice. Mice were euthanized following tumor volume burden exceeding 1000 mm^3^ or > 15 mm in any dimension. The Kaplan–Meier survival curve was plotted at the end of the study using GraphPad Prism 10.

### Isolation of T cells from NSG mouse spleens

At the end of *in vivo* studies, surviving mice were euthanized via CO_2_ asphyxiation and confirmatory cervical dislocation. Spleens were removed via surgical incision and separation from surrounding tissue and transferred to a 50 mL tube containing 5 mL of sterile PBS. Spleens were mechanically dissociated and passed through a 70‐μm cell strainer to generate a single‐cell suspension. The splenocytes were then centrifuged at 453 × *g* for 5 min and resuspended in 100 μL of red blood cell lysis buffer. Cells were incubated for 1 min, then topped up with flow wash buffer and centrifuged for 453 × *g* for 5 min. The supernatant was discarded, and cells were stained for analysis by flow cytometry.

### Statistical analysis

All experiments were performed with three independent healthy donors unless otherwise stated. Data are displayed as mean ± SEM. All statistical tests were performed using GraphPad Prism 10 (GraphPad, San Diego). Statistical tests that returned values <0.05 were considered statistically significant (*P < 0.05, **P < 0.01, ***P < 0.001, ****P < 0.0001).

## AUTHOR CONTRIBUTIONS

**Samuel W.J Smith‐Bell:** Conceptualization; investigation; writing – original draft; methodology; writing – review and editing; visualization; validation; formal analysis; data curation. **Joshua C. Halpin:** Conceptualization; investigation; writing – original draft; writing – review and editing; data curation; formal analysis; visualization; validation; methodology. **Phillip K. Darcy:** Funding acquisition; supervision; resources. **Thiloma Liyanage:** Investigation; data curation. **Lachlan J. Dobson:** Writing – original draft; writing – review and editing; investigation; data curation. **Alexander D. McLellan:** Conceptualization; funding acquisition; writing – original draft; writing – review and editing; methodology; project administration; resources; supervision.

## CONFLICT OF INTEREST

The authors have no conflicts of interest to declare.

## Supporting information


Supplementary figure 1.

Supplementary figure 2.

Supplementary figure 3.

Supplementary figure 4.

Supplementary figure 5.

Supplementary Figure 6.

Supplementary figure 7.

Supplementary figure 8.

Supplementary table 1.

Supplementary table 2.

## Data Availability

The data that support the findings of this study are available from the corresponding author upon reasonable request.

## References

[1] Neelapu SS , Locke FL , Bartlett NL , *et al*. Axicabtagene Ciloleucel CAR T‐cell therapy in refractory large B‐cell lymphoma. N Engl J Med 2017; 377: 2531–2544. 29226797 10.1056/NEJMoa1707447 PMC5882485

[2] Maude SL , Laetsch TW , Buechner J , *et al*. Tisagenlecleucel in children and young adults with B‐cell lymphoblastic leukemia. N Engl J Med 2018; 378: 439–448. 29385370 10.1056/NEJMoa1709866 PMC5996391

[3] Prommersberger S , Reiser M , Beckmann J , *et al*. CARAMBA: a first‐in‐human clinical trial with SLAMF7 CAR‐T cells prepared by virus‐free sleeping beauty gene transfer to treat multiple myeloma. Gene Ther 2021; 28: 560–571. 33846552 10.1038/s41434-021-00254-w PMC8455317

[4] Daei Sorkhabi A , Mohamed Khosroshahi L , Sarkesh A , *et al*. The current landscape of CAR T‐cell therapy for solid tumors: mechanisms, research progress, challenges, and counterstrategies. Front Immunol 2023; 14: 1113882. 37020537 10.3389/fimmu.2023.1113882 PMC10067596

[5] Walker AJ , Majzner RG , Zhang L , *et al*. Tumor antigen and receptor densities regulate efficacy of a chimeric antigen receptor targeting anaplastic lymphoma kinase. Mol Ther 2017; 25: 2189–2201. 28676342 10.1016/j.ymthe.2017.06.008 PMC5589087

[6] Stock S *et al*. Mechanisms and strategies for safe chimeric antigen receptor T‐cell activity control. Int J Cancer 2023; 153: 1706–1725. 37350095 10.1002/ijc.34635

[7] Han X , Wang Y , Wei J , Han W . Multi‐antigen‐targeted chimeric antigen receptor T cells for cancer therapy. J Hematol Oncol 2019; 12: 128. 31783889 10.1186/s13045-019-0813-7 PMC6884912

[8] Flugel CL , Majzner RG , Krenciute G , *et al*. Overcoming on‐target, off‐tumour toxicity of CAR T cell therapy for solid tumours. Nat Rev Clin Oncol 2023; 20: 49–62. 36418477 10.1038/s41571-022-00704-3 PMC10278599

[9] Favier B , Burroughs NJ , Wedderburn L , Valitutti S . TCR dynamics on the surface of living T cells. Int Immunol 2001; 13: 1525–1532. 11717193 10.1093/intimm/13.12.1525

[10] Chen H , Xu X , Hu W , *et al*. Self‐programmed dynamics of T cell receptor condensation. Proc Natl Acad Sci U S A 2023; 120: e2217301120. 37399423 10.1073/pnas.2217301120 PMC10334747

[11] Fraessle SP , Tschulik C , Effenberger M , *et al*. Activation‐inducible CAR expression enables precise control over engineered CAR T cell function. Commun Biol 2023; 6: 604. 37277433 10.1038/s42003-023-04978-w PMC10241805

[12] Guo T , Ma D , Lu TK . Sense‐and‐respond payload delivery using a novel antigen‐inducible promoter improves suboptimal CAR‐T activation. ACS Synth Biol 2022; 11: 1440–1453. 35316028 10.1021/acssynbio.1c00236 PMC9016769

[13] Uchibori R , Teruya T , Ido H , *et al*. Functional analysis of an inducible promoter driven by activation signals from a chimeric antigen receptor. Mol Ther Oncolytics 2019; 12: 16–25. 30662937 10.1016/j.omto.2018.11.003 PMC6325072

[14] Liu Y , Di S , Shi B , *et al*. Armored inducible expression of IL‐12 enhances antitumor activity of Glypican‐3‐targeted chimeric antigen receptor‐engineered T cells in hepatocellular carcinoma. J Immunol 2019; 203: 198–207. 31142602 10.4049/jimmunol.1800033

[15] Chmielewski M , Kopecky C , Hombach AA , Abken H . IL‐12 release by engineered T cells expressing chimeric antigen receptors can effectively muster an antigen‐independent macrophage response on tumor cells that have shut down tumor antigen expression. Cancer Res 2011; 71: 5697–5706. 21742772 10.1158/0008-5472.CAN-11-0103

[16] Zhang L , Kerkar SP , Yu Z , *et al*. Improving adoptive T cell therapy by targeting and controlling IL‐12 expression to the tumor environment. Mol Ther 2011; 19: 751–759. 21285960 10.1038/mt.2010.313 PMC3070103

[17] Mazinani M , Rahbarizadeh F . CAR‐T cell potency: from structural elements to vector backbone components. Biomarker Res 2022; 10: 70. 10.1186/s40364-022-00417-w PMC9487061 36123710

[18] Caruso HG , Heimberger AB , Cooper LJN . Steering CAR T cells to distinguish friend from foe. Onco Targets Ther 2019; 8: e1271857. 10.1080/2162402X.2016.1271857 PMC6791456 31646067

[19] Caruso HG , Hurton LV , Najjar A , *et al*. Tuning sensitivity of CAR to EGFR density limits recognition of Normal tissue while maintaining potent antitumor activity. Cancer Res 2015; 75: 3505–3518. 26330164 10.1158/0008-5472.CAN-15-0139 PMC4624228

[20] Ho JY , Wang L , Liu Y , *et al*. Promoter usage regulating the surface density of CAR molecules may modulate the kinetics of CAR‐T cells in vivo. Mol Ther Methods Clin Dev 2021; 21: 237–246. 33869653 10.1016/j.omtm.2021.03.007 PMC8027690

[21] Rad SMA , Poudel A , Tan GMY , McLellan AD . Promoter choice: who should drive the CAR in T cells? PLoS One 2020; 15: e0232915. 32706785 10.1371/journal.pone.0232915 PMC7380635

[22] Chen X , Khericha M , Lakhani A , *et al*. Rational tuning of CAR tonic signaling yields superior T‐cell therapy for cancer. *bioRxiv*, 2020.2010.2001.322990. 2020.

[23] Long AH , Haso WM , Shern JF , *et al*. 4‐1BB costimulation ameliorates T cell exhaustion induced by tonic signaling of chimeric antigen receptors. Nat Med 2015; 21: 581–590. 25939063 10.1038/nm.3838 PMC4458184

[24] Landoni E , Fucá G , Wang J , *et al*. Modifications to the framework regions eliminate chimeric antigen receptor tonic signaling. Cancer Immunol Res 2021; 9: 441–453. 33547226 10.1158/2326-6066.CIR-20-0451 PMC8137530

[25] Pietrobon V , Todd LA , Goswami A , Stefanson O , Yang Z , Marincola F . Improving CAR T‐cell persistence. Int J Mol Sci 2021; 22: 10828. 34639168 10.3390/ijms221910828 PMC8509430

[26] Gumber D , Wang LD . Improving CAR‐T immunotherapy: overcoming the challenges of T cell exhaustion. EBioMedicine 2022; 77: 103941. 35301179 10.1016/j.ebiom.2022.103941 PMC8927848

[27] Majzner RG , Rietberg SP , Sotillo E , *et al*. Tuning the antigen density requirement for CAR T‐cell activity. Cancer Discov 2020; 10: 702–723. 32193224 10.1158/2159-8290.CD-19-0945 PMC7939454

[28] Rodriguez‐Marquez P , Calleja‐Cervantes ME , Serrano G , *et al*. CAR density influences antitumoral efficacy of BCMA CAR T cells and correlates with clinical outcome. Sci Adv 2022; 8: eabo0514. 36179026 10.1126/sciadv.abo0514 PMC9524842

[29] Odagiu L , May J , Boulet S , Baldwin TA , Labrecque N . Role of the orphan nuclear receptor NR4A family in T‐cell biology. Front Endocrinol (Lausanne) 2020; 11: 624122. 33597928 10.3389/fendo.2020.624122 PMC7883379

[30] Abdou HS , Robert NM , Tremblay JJ . Calcium‐dependent Nr4a1 expression in mouse Leydig cells requires distinct AP1/CRE and MEF2 elements. J Mol Endocrinol 2016; 56: 151–161. 26647388 10.1530/JME-15-0202

[31] Chen AXY , Yap KM , Kim JS , *et al*. Rewiring endogenous genes in CAR T cells for tumour‐restricted payload delivery. Nature 2025; 644: 241–251. 40604285 10.1038/s41586-025-09212-7 PMC12328239

[32] Messeguer X , Escudero R , Farré D , Núñez O , Martínez J , Albà MM . PROMO: detection of known transcription regulatory elements using species‐tailored searches. Bioinformatics 2002; 18: 333–334. 11847087 10.1093/bioinformatics/18.2.333

[33] Greenshpan Y , Sharabi O , Yegodayev KM , *et al*. The contribution of the minimal promoter element to the activity of synthetic promoters mediating CAR expression in the tumor microenvironment. Int J Mol Sci 2022; 23: 7431. 35806439 10.3390/ijms23137431 PMC9266962

[34] Tao Z , Chyra Z , Kotulová J , *et al*. Impact of T cell characteristics on CAR‐T cell therapy in hematological malignancies. Blood Cancer J 2024; 14: 213. 39627220 10.1038/s41408-024-01193-6 PMC11615218

[35] Kouro T , Himuro H , Sasada T . Exhaustion of CAR T cells: potential causes and solutions. J Transl Med 2022; 20: 239. 35606821 10.1186/s12967-022-03442-3 PMC9125881

[36] Tang L , Zhang Y , Hu Y , Mei H . T cell exhaustion and CAR‐T immunotherapy in hematological malignancies. Biomed Res Int 2021; 2021: 6616391. 33728333 10.1155/2021/6616391 PMC7936901

[37] Eyquem J , Mansilla‐Soto J , Giavridis T , *et al*. Targeting a CAR to the TRAC locus with CRISPR/Cas9 enhances tumour rejection. Nature 2017; 543: 113–117. 28225754 10.1038/nature21405 PMC5558614

[38] Tommasi A , Cappabianca D , Bugel M , *et al*. Efficient nonviral integration of large transgenes into human T cells using Cas9‐CLIPT. Mol Ther Methods Clin Dev 2025; 33: 101437. 40123742 10.1016/j.omtm.2025.101437 PMC11930092

[39] Pandey S *et al*. Efficient site‐specific integration of large genes in mammalian cells via continuously evolved recombinases and prime editing. Nat Biomed Eng 2025; 9: 22–39. 38858586 10.1038/s41551-024-01227-1 PMC11754103

[40] Singh N , Perazzelli J , Grupp SA , Barrett DM . Early memory phenotypes drive T cell proliferation in patients with pediatric malignancies. Sci Transl Med 2016; 8: 320ra323. 10.1126/scitranslmed.aad5222 26738796

[41] Hao J , Li R , Zhao X , *et al*. NR4A1 transcriptionally regulates the differentiation of stem‐like CD8+ T cells in the tumor microenvironment. Cell Rep 2024; 43: 114301. 38823016 10.1016/j.celrep.2024.114301

[42] Liu X , Wang Y , Lu H , *et al*. Genome‐wide analysis identifies NR4A1 as a key mediator of T cell dysfunction. Nature 2019; 567: 525–529. 30814730 10.1038/s41586-019-0979-8 PMC6507425

[43] Nielsen HV , Yang L , Mueller JL , *et al*. Nr4a1 and Nr4a3 redundantly control clonal deletion and contribute to an anergy‐like transcriptome in auto‐reactive thymocytes to impose tolerance in mice. Nat Commun 2025; 16: 784. 39824797 10.1038/s41467-025-55839-5 PMC11742425

[44] Nowyhed HN , Huynh TR , Thomas GD , Blatchley A , Hedrick CC . Cutting edge: the orphan nuclear receptor Nr4a1 regulates CD8+ T cell expansion and effector function through direct repression of Irf4. J Immunol 2015; 195: 3515–3519. 26363057 10.4049/jimmunol.1403027 PMC4592102

[45] Schmidt SF , Larsen BD , Loft A , Mandrup S . Cofactor squelching: artifact or fact? Bioessays 2016; 38: 618–626. 27273739 10.1002/bies.201600034

