# Supplementary figures and images for: Auto‐inducible expression of chimeric antigen receptor T cells using the NR4A1 promoter
Source: Immunol Cell Biol. 2026 Mar 8;104(4):381–95. doi: 10.1111/imcb.70095 (PMC13071125; doi:10.1111/imcb.70095)

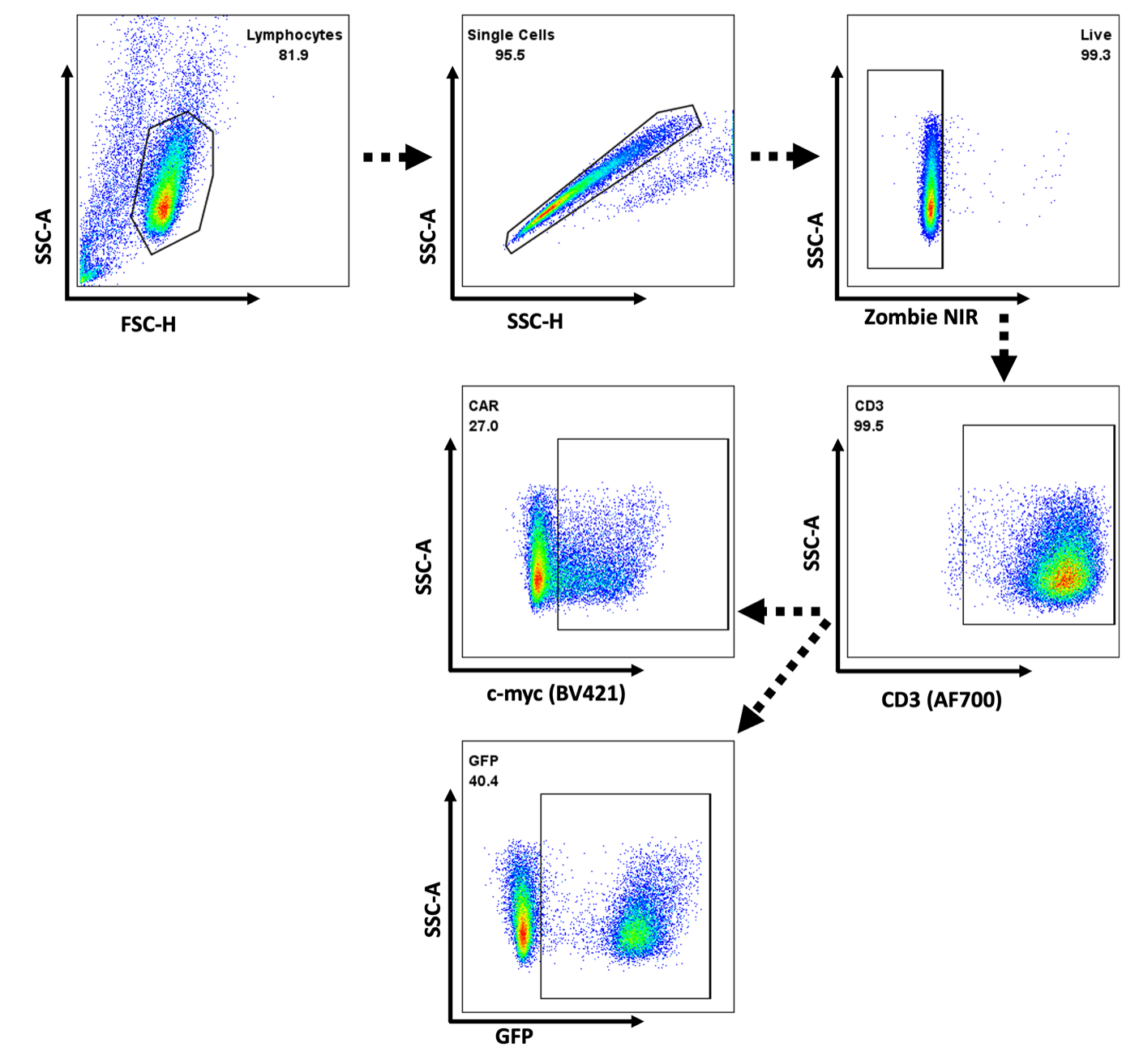

Supplement: Supplementary file 1 — Supplementary figure 1. [file IMCB-104-381-s008.png]

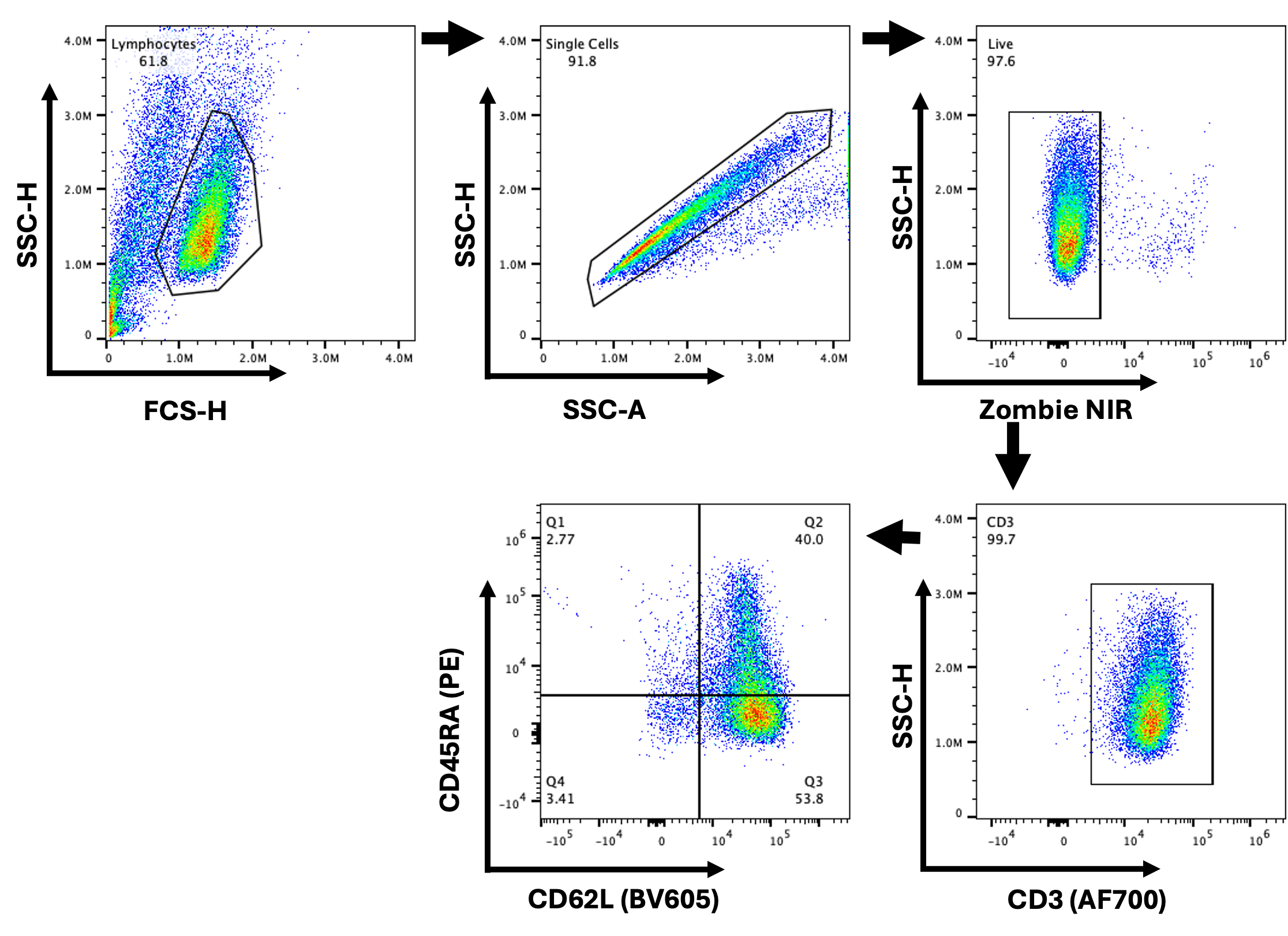

Supplement: Supplementary file 2 — Supplementary figure 2. [file IMCB-104-381-s004.png]

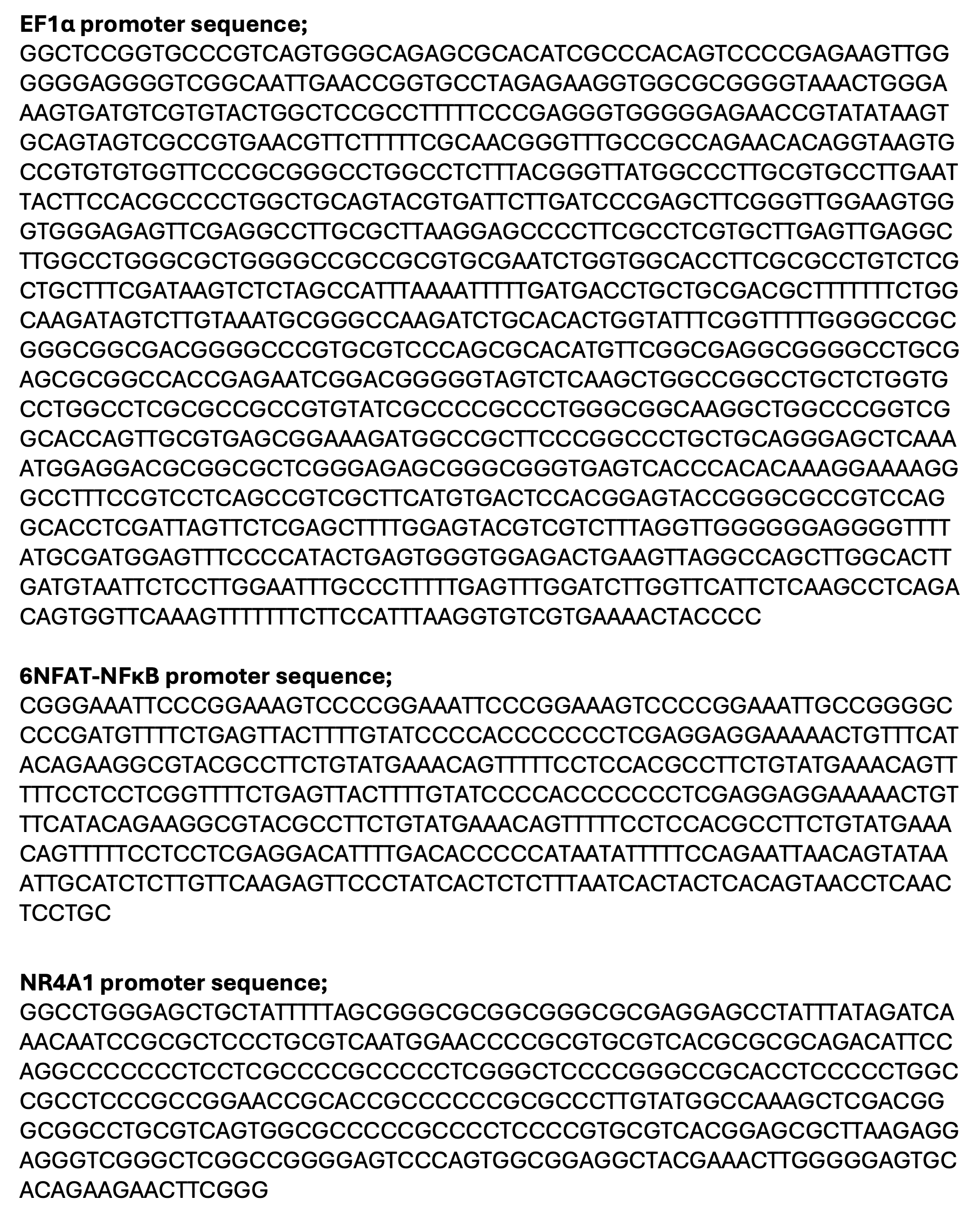

Supplement: Supplementary file 3 — Supplementary figure 3. [file IMCB-104-381-s005.png]

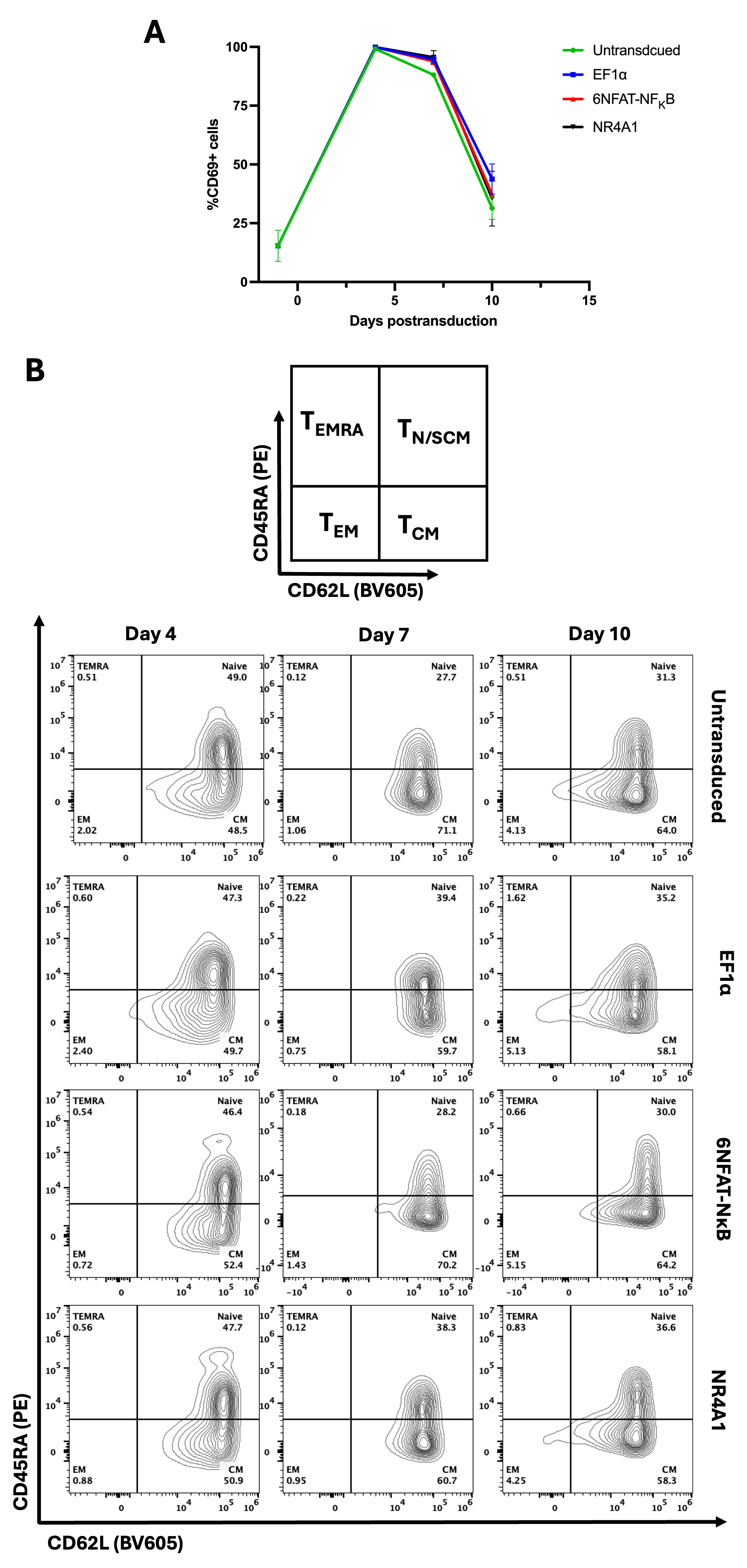

Supplement: Supplementary file 4 — Supplementary figure 4. [file IMCB-104-381-s010.png]

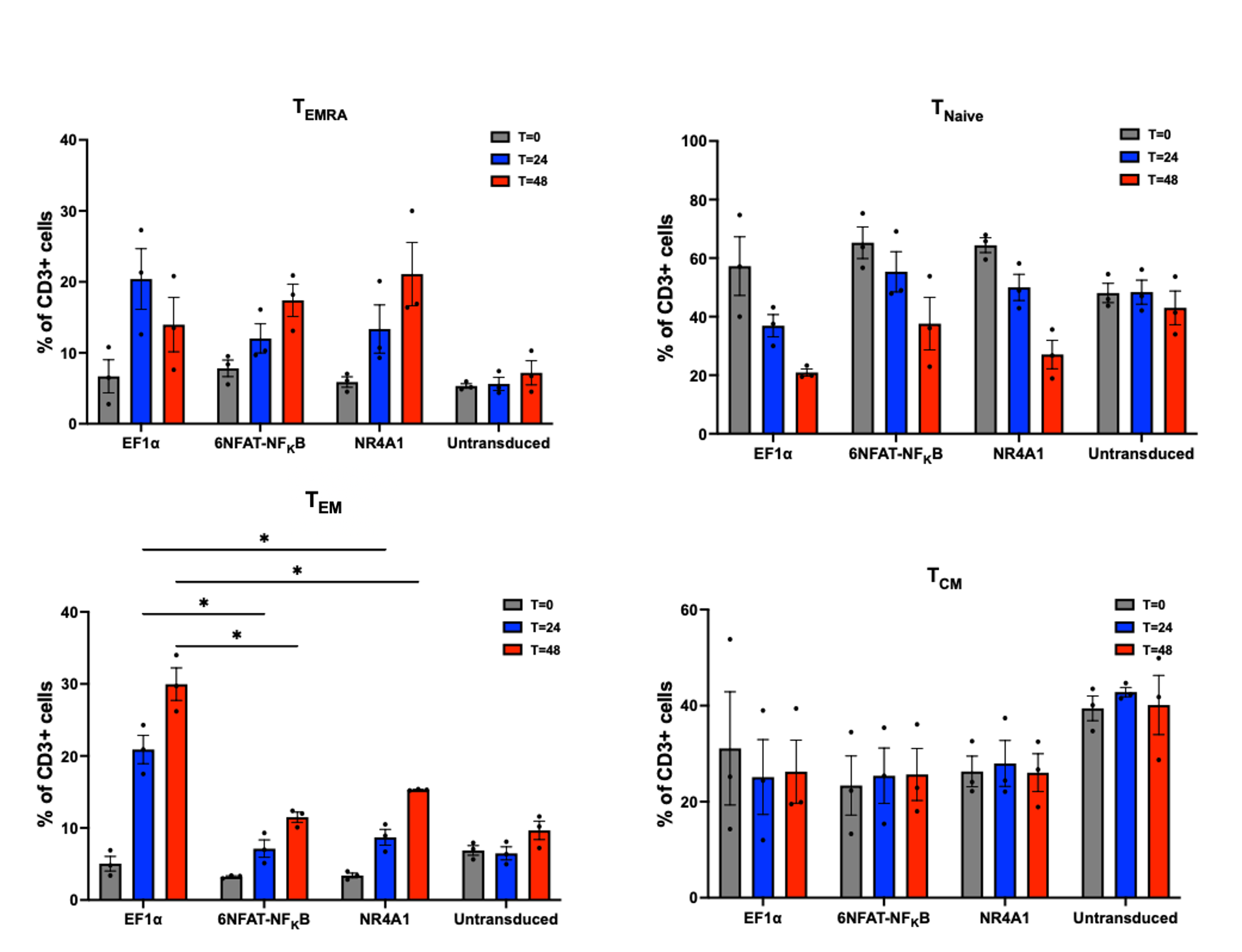

Supplement: Supplementary file 5 — Supplementary figure 5. [file IMCB-104-381-s003.png]

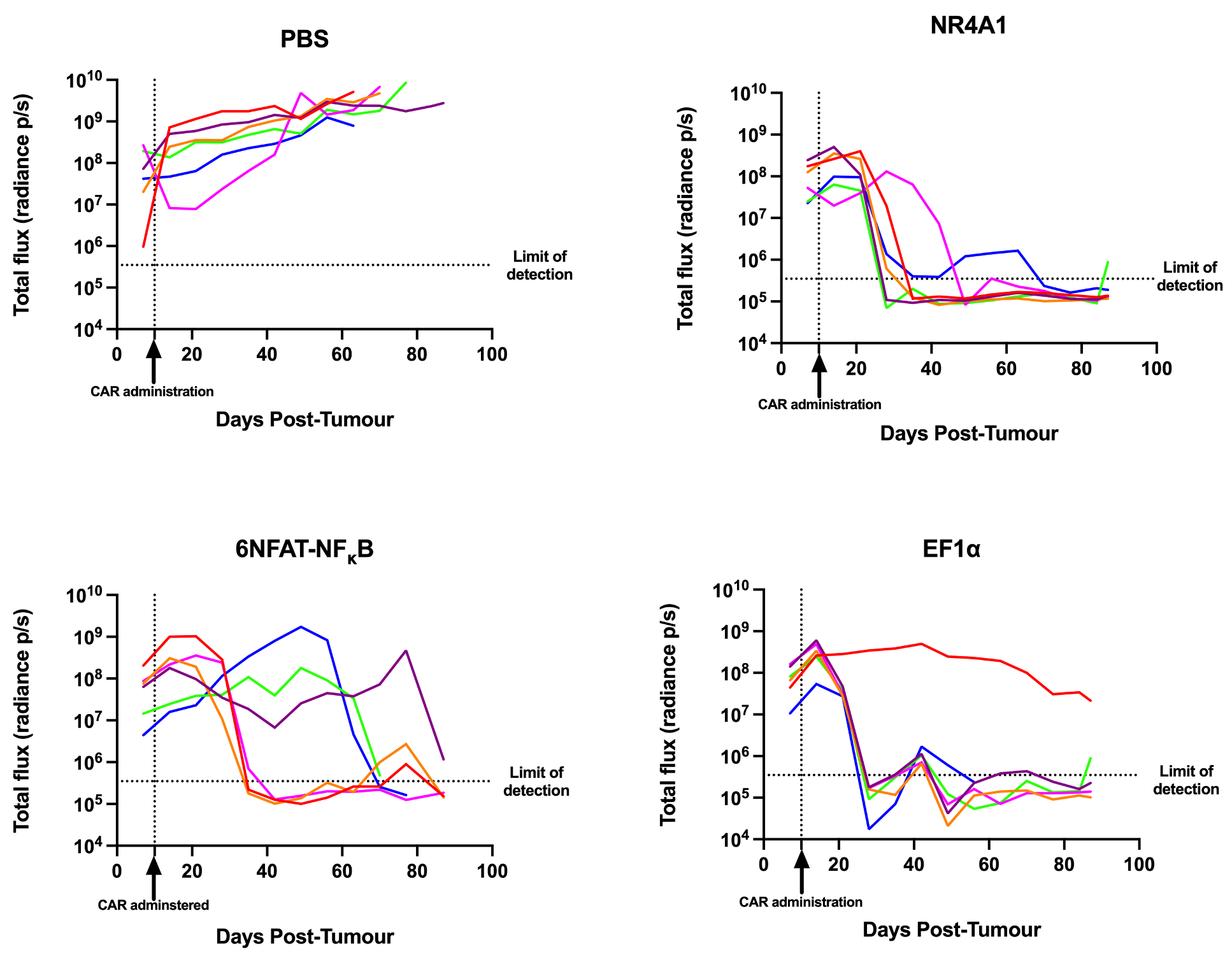

Supplement: Supplementary file 6 — Supplementary Figure 6. [file IMCB-104-381-s001.png]

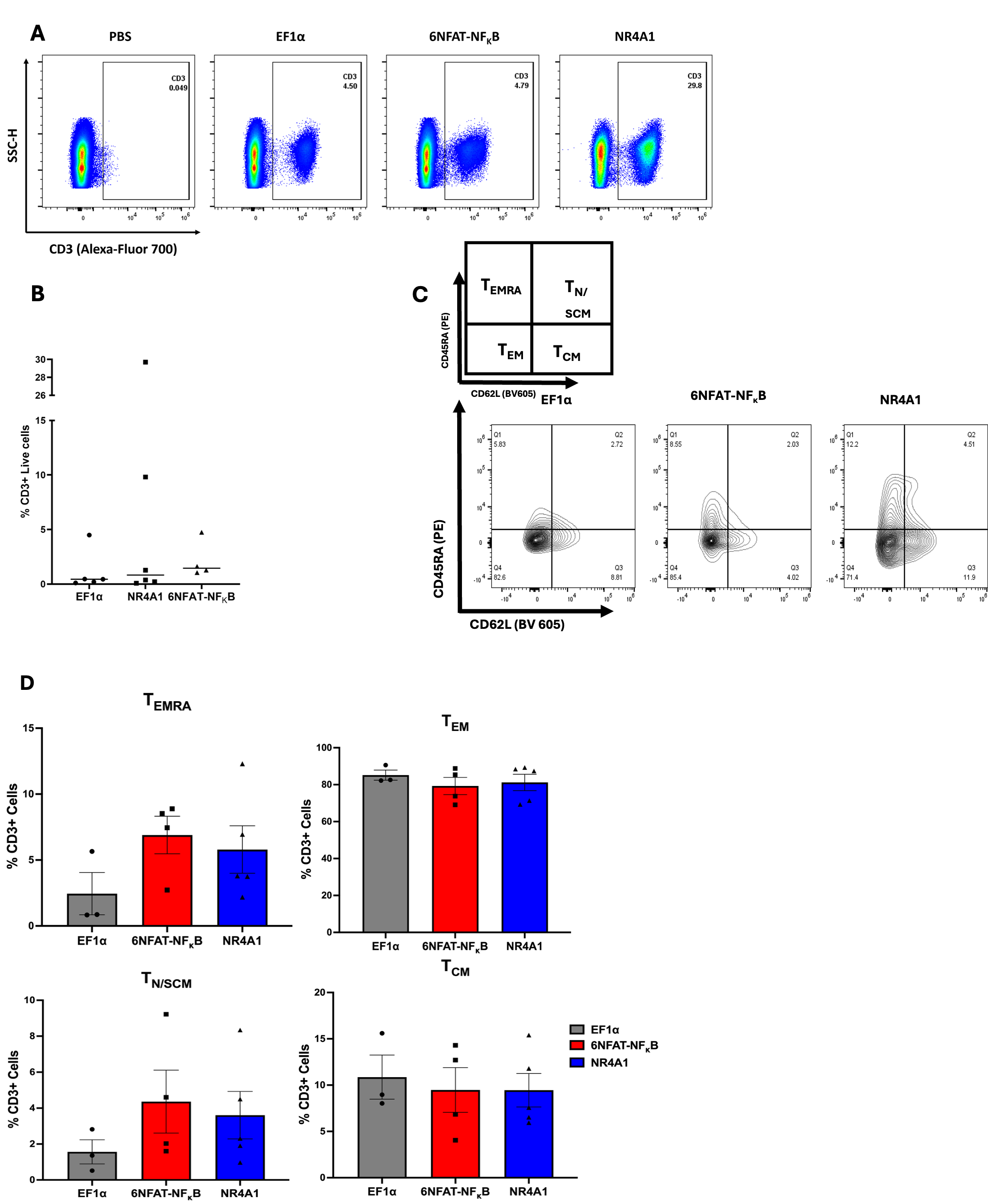

Supplement: Supplementary file 7 — Supplementary figure 7. [file IMCB-104-381-s009.png]

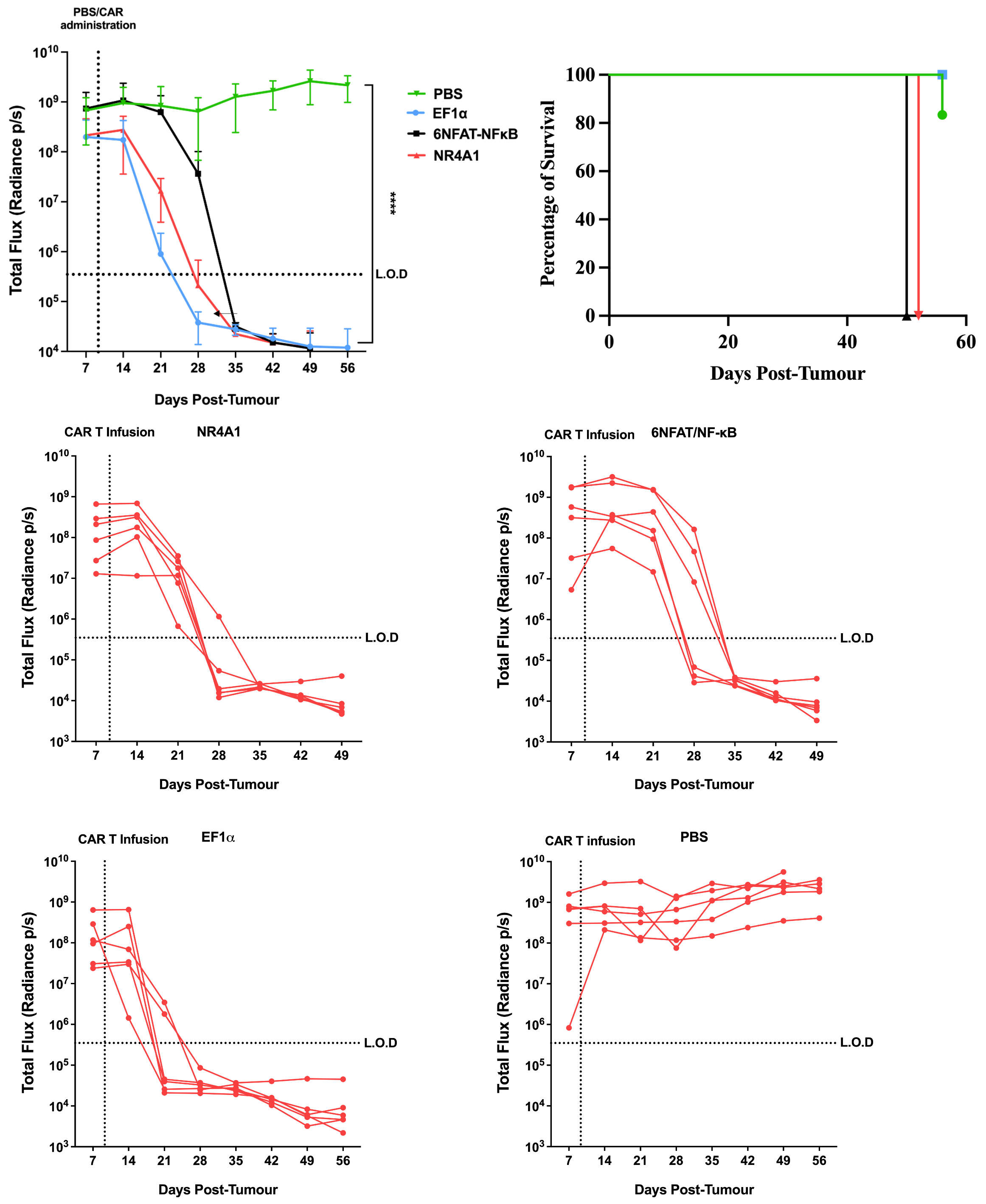

Supplement: Supplementary file 8 — Supplementary figure 8. [file IMCB-104-381-s006.png]
