# Supplementary material for: Auto‐inducible expression of chimeric antigen receptor T cells using the NR4A1 promoter
Source: Immunol Cell Biol. 2026 Mar 8;104(4):381–95. doi: 10.1111/imcb.70095 (PMC13071125; doi:10.1111/imcb.70095)
Supplement: Supplementary file 9 — Supplementary table 1. [file IMCB-104-381-s007.docx]

| **Supplementary Table 1: Antibodies/Reagents used for staining for flow cytometric analysis** | | | | |
| --- | --- | --- | --- | --- |
| **Target (clone) / Reagent** | **Conjugation** | **Isotype** | **Concentration** | **Catalogue number** |
| **CD3 (UCHT1)** | AF700 | Mouse IgG1 | 2.5 μg/mL | BioLegend #300424 |
| **Zombie NIR (N/A)** | Fixable viability dye | N/A | 1/16000  dilution | BioLegend #423106 |
| **cmyc (9E10)** | Biotin | Mouse IgG1 | 2 μg/mL | BioLegend #908805 |
| **Streptavidin** | ΒV421 | N/A | 1 μg/mL | BioLegend #405225 |
| **CD45RA (HI100)** | PE | Mouse IgG2b | 2 μg/mL | BD Pharmigen #555489 |
| **CD62L (DREG-56)** | BV605 | Mouse IgG1 | 2 μg/mL | BD Horizon #562719 |
| **PD1 (EH12.2H7)** | PE Dazzle 594 | Mouse IgG1 | 2 μg/mL | BioLegend #329939 |
| **ΤΙΜ3 (F38-2E2)** | BV650 | Mouse IgG1 | 2 μg/mL | BioLegend #345027 |
| **CD69 (FN50)** | APC | Mouse IgG1 | 2 μg/mL | BioLegend #310910 |
