# Supplementary material for: Auto‐inducible expression of chimeric antigen receptor T cells using the NR4A1 promoter
Source: Immunol Cell Biol. 2026 Mar 8;104(4):381–95. doi: 10.1111/imcb.70095 (PMC13071125; doi:10.1111/imcb.70095)
Supplement: Supplementary file 10 — Supplementary table 2. [file IMCB-104-381-s002.docx]

| ***Supplementary table 2: Primers used to clone AI expression cassettes*** | | |
| --- | --- | --- |
| **Primer name** | Annealing temp (°C) | Sequence (Primer binding region in bold) |
| **6NFAT-Gib-Fwd** | 76.5 | AGCCTCGAGAAGCTTGATCAGCTACCCGGGAAATTCCC |
| **6NFAT-Gib-Rev** | 74.9 | TCGCCCTTGCTCAACATGGTGGCCTCAGAGGCCAG |
| **NR4A1-Gib-Fwd** | 73.8 | AGCCTCGAGAAGCTTGATGGCCTGGGAGCTGCTATTTT |
| **NR4A1-Gib-Rev** | 76.5 | TCGCCCTTGCTCAACATGGTGGCCTCAGAGGCCAG |
| **NR4-Gib-Fwd** | 72.7 | AGCCTCGAGAAGCTTGATCCGCGGAGGAAAAACTGTTT |
| **NR4-Gib-Rev** | 76.1 | TCGCCCTTGCTCAACATGGTGGCTCTAGAGCTCGGTC |
